# Bidirectional two-sample Mendelian randomization analysis identifies causal associations between cardiovascular diseases and frozen shoulder

**DOI:** 10.1186/s13018-024-04600-7

**Published:** 2024-02-03

**Authors:** WeiSong Lu, Bin Pu, Sen Wang, MengZe Li, Yue An, Jie Lian, YongQuan Wang

**Affiliations:** 1 Luzhou Hospital of Traditional Chinese Medicine, Luzhou, Sichuan China; 2 Guangzhou University of Chinese Medicine, Guangzhou, Guangdong China

**Keywords:** Frozen shoulder, Cardiovascular diseases, Mendelian randomization, Genome-wide association studies

## Abstract

**Background:**

Although prior observational studies indicate an association between cardiovascular diseases (CVDs) and frozen shoulder (FS), the potential causal relationship between them remains uncertain. This study aims to explore the genetic causal relationship between CVDs and FS using Mendelian randomization (MR).

**Methods:**

Genetic variations closely associated with FS were obtained from the FinnGen Consortium. Summary data for CVD, including atrial fibrillation (AF), coronary artery disease (CAD), heart failure (HF), myocardial infarction (MI), stroke, and ischemic stroke (IS), were sourced from several large-scale genome-wide association studies (GWAS). MR analysis was performed using inverse variance weighting (IVW), MR Egger, and weighted median methods. IVW, as the primary MR analysis method, complemented by other sensitivity analyses, was utilized to validate the robustness of the results. Further reverse MR analysis was conducted to explore the presence of reverse causal relationships.

**Results:**

In the forward MR analysis, genetically determined risk of stroke and IS was positively associated with FS (OR [95% CI] = 1.58 (1.23–2.03), *P* < 0.01; OR [95% CI] = 1.46 (1.16–1.85), *P* < 0.01, respectively). There was no strong evidence of an effect of genetically predicted other CVDs on FS risk. Sensitivity analyses confirmed the robustness of the results. In the reverse MR analysis, no causal relationships were observed between FS and various CVDs.

**Conclusion:**

The study suggests that stroke increases the risk of developing FS. However, further basic and clinical research is needed to substantiate our findings.

**Supplementary Information:**

The online version contains supplementary material available at 10.1186/s13018-024-04600-7.

## Introduction

Frozen shoulder (FS) is a fibroproliferative disorder characterized by pain and progressive restriction of shoulder motion [1]. Despite its self-limiting nature, with spontaneous recovery typically occurring within 1–2 years, research suggested that numerous FS-related symptoms could persist in 20–50% of patients, encompassing stiffness and pain [2, 3]. The treatment of FS emphasizes physical therapy, pharmacological relief, and potential surgery. Meanwhile, preventive measures are crucial for alleviating patient symptoms and reducing the socio-medical burden [4]. Certain preventive measures can regulate the occurrence and progression of FS; thus, identifying novel FS risk factors and interventions is imperative.

Cardiovascular diseases (CVDs), primarily encompassing coronary artery disease (CAD), heart failure (HF), myocardial infarction (MI), and stroke, have emerged as the leading causes of morbidity and mortality in the global population [5]. World Health Organization statistics indicated an annual death toll of about 17.8 million due to CVD, contributing to roughly 30% of the total global fatalities [6]. Recent studies have demonstrated a close association between various CVDs and FS. A clinical retrospective study involving 262 FS patients reported that the overall risk of FS was more than three times higher in CAD patients [7]. FS patients have a 1.22 times higher risk of experiencing a stroke compared to the healthy control group [8]. Similarly, a large cohort study indicated that FS patients face an elevated risk of MI compared to the general population [9]. Overall, the existing findings in the observational studies section demonstrate the correlation between CVDs and FS. Despite the association, the mentioned studies have not adequately distinguished the causal relationship between CVDs and FS, nor have they addressed the evidence gap that may result from unmeasured confounding or reverse causation. Therefore, caution is still needed in interpreting these relationships.

Observational studies face limitations in establishing causality due to the inability to control interventions, confounding, and bias. In recent years, in order to overcome the limitations of observational studies, Mendelian randomization (MR) has emerged as a novel approach to explore causal association between etiology and diseases [10, 11]. Based on the genome-wide association study (GWAS), MR uses natural genetic variation to simulate randomized controlled trials (RCTs) and uses genetic variation as instrumental variables (IVs) to assess the causal relationship between exposures and outcomes. In MR studies, single-nucleotide polymorphisms (SNPs) are utilized as IVs to estimate the causal associations between exposures and target outcomes [12]. These SNPs adhere to the random distribution principle of genetic variation, effectively mitigating the potential impacts of confounding factors and reverse causality, given that genetic variations manifest prior to the onset of the disease [13]. Meanwhile, the advancing GWASs have furnished robust and dependable IV support for MR study.

Although Lv et al. have reported the causal relationship between CVD and FS using MR methods, their study was limited to the association between stroke and FS and did not analyze the reverse causal relationship between them [14]. Therefore, the current study employs newly released GWAS data to conduct a two-sample bidirectional MR study, aiming to investigate the presence of a direct causal association between multiple CVDs and FS, providing a theoretical basis for clinical practice. To our knowledge, this is the first two-sample MR study endeavoring to unveil the causality between multiple CVDs and FS. By circumventing certain limitations of observational studies in the process of causal inference, this study holds the potential to offer a clearer perspective for us to delve deeper into understanding this association.

## Methods

### Data source

In order to mitigate potential confounding bias arising from racial stratification, our study exclusively focuses on participants of European descent to ensure the reliability and consistency of outcomes.

Aggregated statistical data for FS were obtained from FinnGen, encompassing a total of 170,583 Europeans (2942 cases and 167,641 controls). FinnGen constitutes a large-scale public–private partnership aimed at gathering and analyzing genomic and health data from 500,000 Finnish biobank participants. SNPs were analyzed using a mixed-model logistic regression, adjusting for gender, age, 10 principal components, genetic relatedness, and genotyping batch. Further comprehensive information regarding FinnGen can be accessed on its official website [https://www.finngen.fi/en].

We extracted genetic association data related to CVDs from five large-scale meta-analyses. Data on atrial fibrillation (AF) were derived from a GWAS of susceptibility genes published in 2018, the study included 60,620 patients with atrial fibrillation, 970,216 healthy controls and revealed 142 independent risk variants at 111 loci and prioritized 151 functional candidate genes likely to be involved in AF [15]. This database compares data from six cohort studies (the Nord-Trøndelag Health Study, the Michigan Genomics Initiative, deCODE, DiscovEHR, AFGen Consortium, and the UK Biobank). The GWAS of CAD data from the CARDIoGRAMplusC4D consortium and UK Biobank includes 122,733 cases and 424,528 controls [16]. Data on HF compilation included 47,309 cases and 930,014 controls from 26 cohorts of European ancestry, showing 12 independent variants at 11 genomic loci were associated with HF. The definition of HF was based on the Heart Failure Molecular Epidemiology (HERMES) Consortium targeting therapeutic endpoints [17]. The MI GWAS dataset originated from another GWAS analysis, including 14,825 cases and 44,000 controls of European ancestry [18]. The summary statistical data for stroke were derived from the MEGASTROKE consortium, which comprised 446,696 participants of European ancestry (40,585 stroke cases and 406,111 controls). Among these stroke cases, 34,217 individuals were classified as suffering from ischemic stroke (IS) [19]. Within this study, the definition of strokes is in accordance with guidelines from the World Health Organization (WHO). Detailed information regarding data sources and definitions is presented in Table 1.

**Table 1:** GWAS summary statistics: source and description

GWAS ID	Phenotypes	Year	Author or consortium	Population	Sample size		PubmedID
					Cases	Controls	
ebi-a-GCST006414	AF	2018	Nielsen JB	European	60,620	970,216	30,061,737
ebi-a-GCST005195	CAD	2017	Van der Harst P	European	122,733	424,528	29,212,778
ebi-a-GCST009541	HF	2020	Shah S	European	47,309	930,014	31,919,418
ebi-a-GCST011365	MI	2021	Hartiala JA	European	14,825	44,000	33,532,862
ebi-a-GCST005838	Stroke	2018	Malik R	European	40,585	406,111	29,531,354
ebi-a-GCST005843	IS	2018	Malik R	European	34,217	406,111	29,531,354
finn-b-M13_ADHCAPSULITIS	FS	2021	FinnGen	European	2942	167,641	NA

*AF* atrial fibrillation; *CAD* coronary artery disease; *HF* heart failure; *MI* myocardial infarction; *IS* ischemic stroke; *FS* frozen shoulder

The involved published GWAS meta-analyses and the FinnGen study have obtained approvals from relevant ethical review bodies and have received informed consent from participants. Our study solely analyzes publicly available summary-level statistical data; thus, new ethical review committee approval is unnecessary.

### MR assumptions and genetic instrument selection

**Fig. 1:**
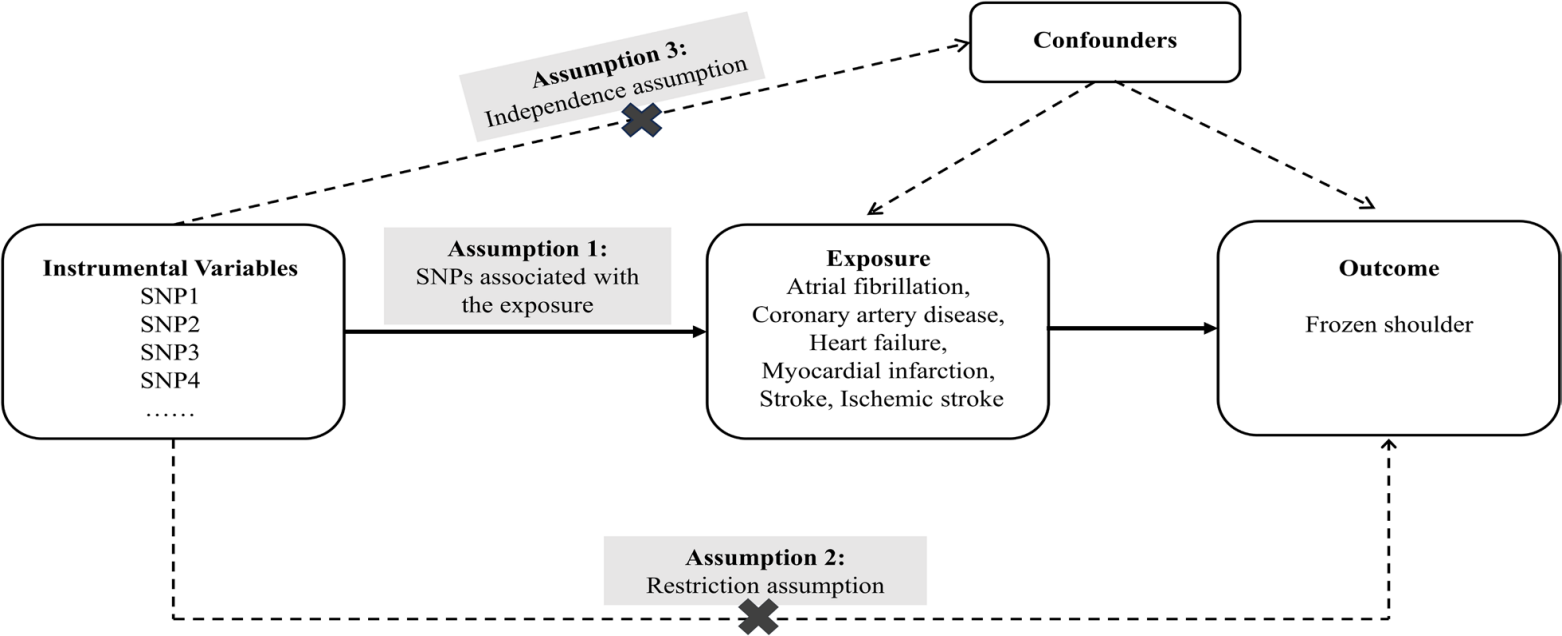
Scatter plots for causal effect of CVDs on frozen shoulder

Genetic variations serve as tools for establishing causal association in MR analysis. To attain unbiased estimates of the association between CVD and FS-related features, the following conditions must be met: (1) The selected instrumental variable (IV) is exclusively linked to CVD; (2) The eligible exposure IV remains independent of any confounding factors; (3) The IV solely affects FS through CVD (Fig. 1). With a genome-wide significance threshold set at *P* < 5.0 × 10^–8^, we selected potential IVs from each SNP associated with CVD. Based on genome-wide significance, to ensure independence among the utilized IVs, we set a linkage disequilibrium (LD) threshold of *R*^2^ < 0.001 and employed a window size of 1000 kb.

In cases where exposure-associated SNPs are absent in the outcome dataset, we employed alternative SNPs significantly correlated with the SNPs of interest (*R*^2^ > 0.8). If suitable replacement SNPs were not available, the SNP was discarded. Simultaneously, we set a minimum allele frequency (MAF) of 0.3 to ensure SNP commonality. To eliminate bias, we harmonized effect alleles between the exposure and outcome datasets and excluded all SNPs with palindromic characteristics. Weak IVs typically lack strong correlation with the exposure factor, leading to diminished effectiveness in explaining the genetic variation in the exposure. The presence of weak IVs results in an augmentation of bias between estimated and actual values. To quantify IV strength, we utilize the F-statistic, where an F-statistic > 10 is considered to indicate sufficient instrument strength [20]. The formula for calculating the F-statistic is as follows: **F** = ((N − 2) × *R*^2^ / (1 − *R*^2^)), where *R*^2^ signifies the extent to which the SNP explains the exposure, and N denotes the sample size [20]. The calculation formula for *R*^2^ is as follows: *R*^2^ = 2 × MAF × (1—MAF) × β^2^, where MAF refers to the minor allele frequency and *β* represents the effect size of the exposure [21]. In the absence of MAF, we utilize the formula *R*^2^ = β^2^/(*β*^2^ + SE^2^ × N)to compute *R*^2^ [22].

### Statistical analysis

In the MR analysis, we employed the inverse variance weighted (IVW), weighted median (WM), and MR Egger methods. Compared to the conventional fixed-effect IVW method, the random-effects IVW method demonstrates enhanced robustness in the presence of instrument selection heterogeneity, resulting in more conservative and realistic parameter estimates [23] (Additional file 1: Table S1). Given this, we consider the results of the IVW method as the basis for primary outcome assessment. To enhance result reliability, we stipulated that the IVW results should be statistically significant at minimum, and the outcomes of WM and MR-Egger methods must align directionally with the IVW results. To control for the type I error rate, the Benjamini–Hochberg method was used to adjust for multiple testing. The false discovery rate (FDR) threshold was set at 0.05 for significance.

Following the MR analysis, we utilized Cochran's Q test to evaluate heterogeneity in the effect of CVD-related SNPs on FS risk [24]. When the test results indicate heterogeneity (*P* < 0.05), we introduce the MR-PRESSO method to remove IVs with heterogeneity and then perform a re-analysis of the IVs not identified as heterogeneous [25]. We also employed the MR-Egger intercept method to assess evidence of horizontal pleiotropy among the selected SNPs (presence of horizontal pleiotropy was speculated at P < 0.05) [26]. Moreover, we conducted leave-one-out analysis to verify if any individual SNP significantly influences the outcomes. To assess the reliability and heterogeneity of the estimates, we employed forest plots and funnel plots. Scatter plots were generated to visually present the estimated effect sizes.

In the reverse causal analysis, we replicated the aforementioned methodology, employing an SNP set associated with FS to explore the causal impact of FS on CVDs. It should be noted that given the restricted count of SNPs closely associated with FS (P < 5 × 10^–8^), we employed a more relaxed threshold (P < 5 × 10^–6^) for SNP identification [27, 28].

“Two SampleMR” (version 0.5.6) of R software (version 4.2.1) was used for all analyses. P values less than 0.05 were considered statistically significant. We adhered to the guidance of the STROBE-MR guidelines in reporting the MR study [29].

## Results

### Characteristics of the genetic instruments

Adhering to established stringent quality control criteria, we selected for a set of SNPs associated with CVDs and FS to serve as IVs. Specifically, we selected 98 SNPs associated with AF (rs4642101 replaced by rs11717013), 61 SNPs associated with CAD, 9 SNPs associated with HF, 67 SNPs associated with MI (rs10404176, rs112374545, rs12897285, rs180803, rs433903, rs9865841 replaced by rs10423961, rs113722226, rs1958320, rs77036345, rs355788, rs6779146, respectively), 16 SNPs associated with stroke, 17 SNPs associated with IS (rs9909858 replaced by rs60460011), and 12 SNPs associated with FS. The F-statistics for all these genetic variations are above the threshold of 10 (range: 30–45951). Because higher F-statistics indicate stronger instruments, the IVs we selected have a robust power to predict CVD and FS, rendering them suitable for application as IVs in MR analysis. Additional file 1: Table S2 and S3 and Additional file 1: Fig. S1 furnish comprehensive details about these SNPs.

### Causal effects of CVDs on FS

**Fig. 2:**
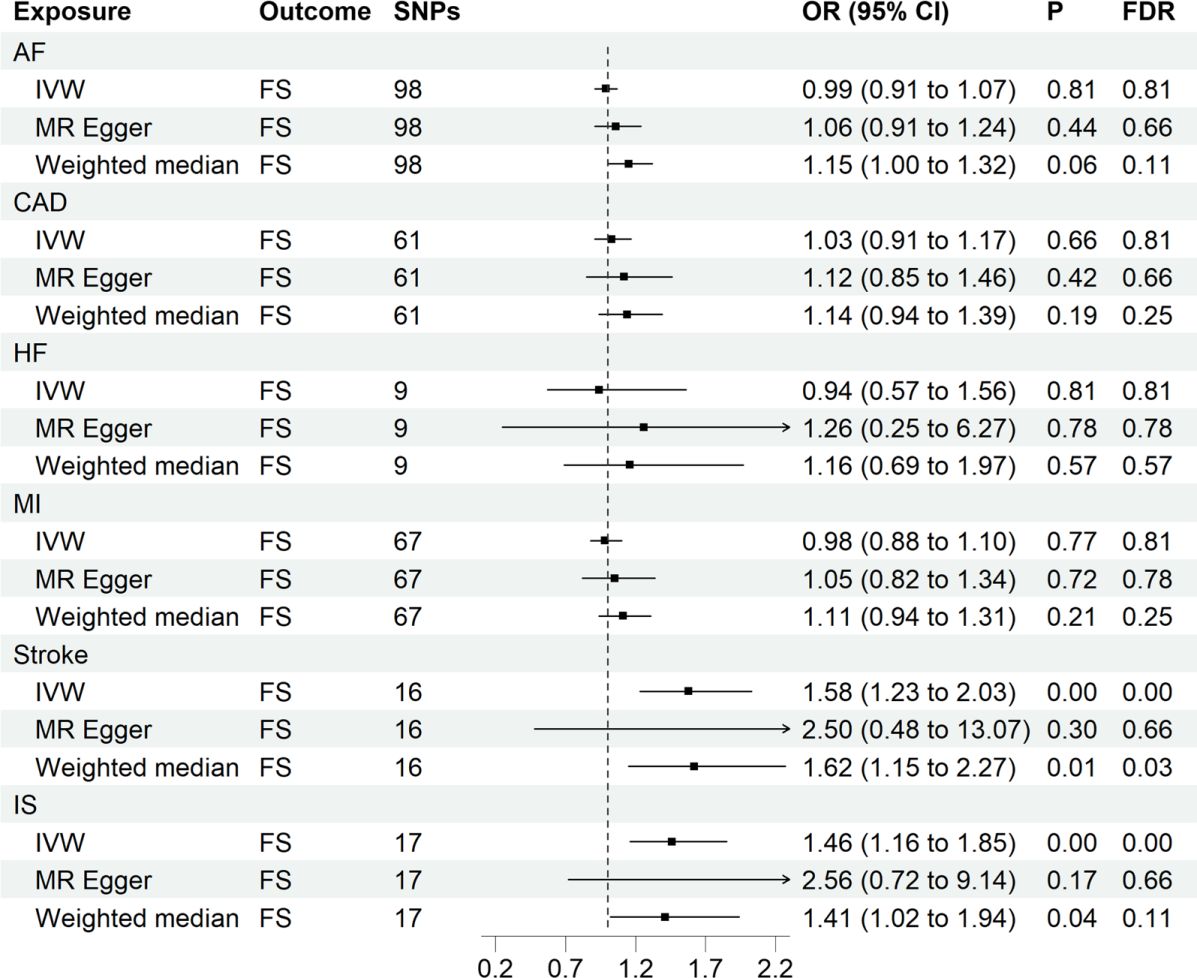
Forest plots of MR study using genetically predicted CVDs with frozen shoulder. IVW, inverse variance weighted; AF, atrial fibrillation; CAD, coronary artery disease; HF, heart failure; MI, myocardial infarction; IS, ischemic stroke. High risk: P  < 0.05 and FDR < 0.05; potential risk: P  < 0.05 and FDR > 0.05; unclear: P  > 0.05; protective: P  < 0.05 and FDR < 0.05

The IVW, MR Egger, and WM were employed to assess the causal association between CVDs and FS. The IVW was employed as the principal method. When conducting the analysis using the IVW, we noted a significant elevation in the risk of FS due to genetic propensity toward stroke and IS (OR = 1.58, *P* < 0.05; OR = 1.46, *P* < 0.05, respectively). Nevertheless, in the causal association analysis between other CVDs (including AF, CAD, HF, and MI) and FS, we did not observe significant results (all *P* > 0.05 & FDR > 0.05). The MR Egger and WM also demonstrated trends resembling IVW, providing additional support for the robustness of the causal relationship inference between FS and CVDs (Fig. 2).

**Fig. 3:**
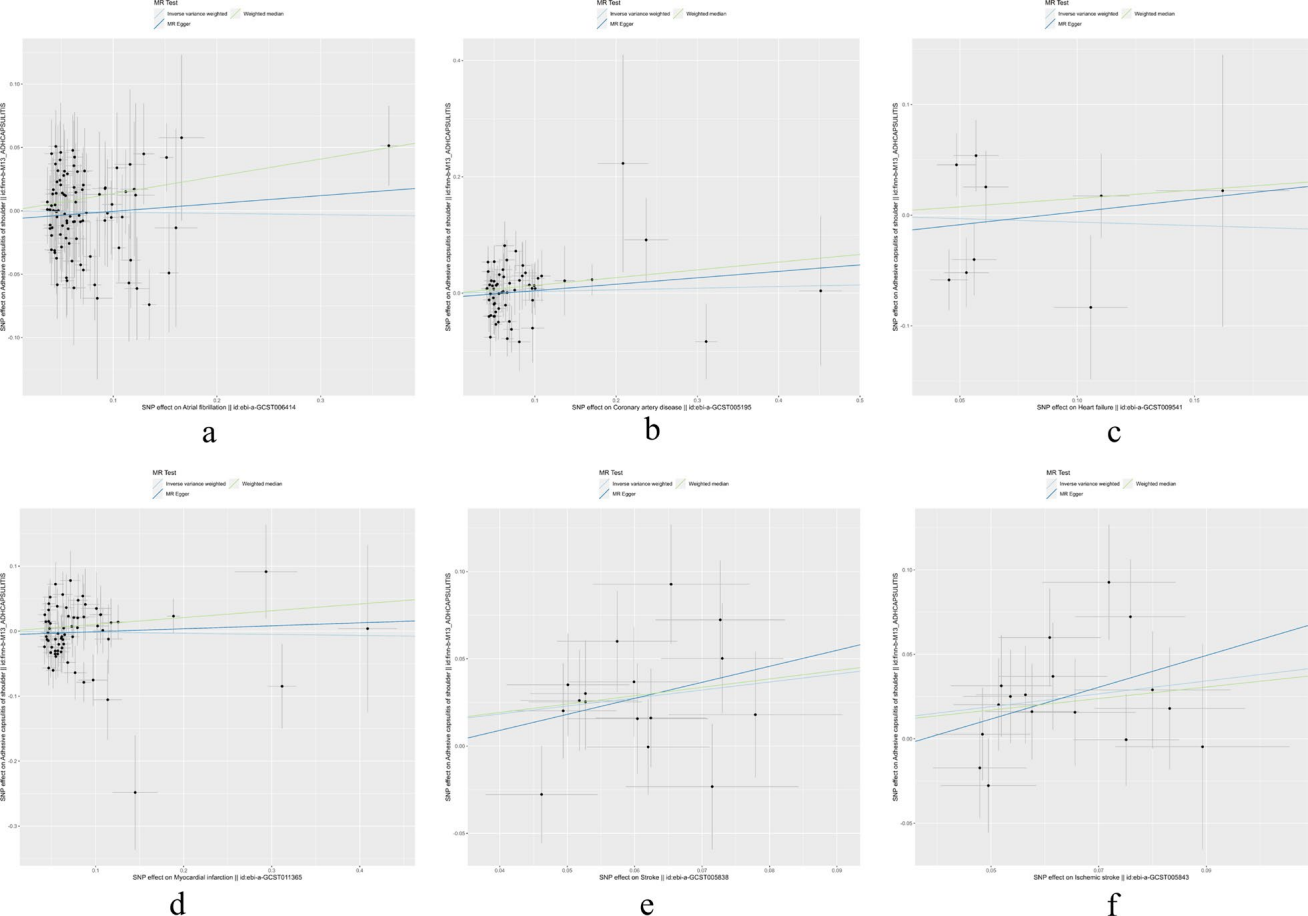
Scatter plots for causal effect of CVDs on frozen shoulder. **a** Atrial fibrillation on frozen shoulder, **b** coronary artery disease on frozen shoulder, **c** heart failure on frozen shoulder, **d** myocardial infarction on frozen shoulder, **e** stroke on frozen shoulder, **f** ischemic stroke on frozen shoulder

Table 2 provides a compilation of detailed results concerning heterogeneity and horizontal pleiotropy. Regarding the MR Egger regression, our analysis findings indicated none of the examined SNPs exhibited signs of horizontal pleiotropy. Simultaneously, while conducting the Cochran's Q test, the P-values for the Q statistics significantly surpassed the threshold of 0.05. This indicated the absence of noteworthy heterogeneity among IVs we employed. To further confirm the robustness of our results, we conducted leave-one-out analysis, and the relevant results are presented in Additional file 1: Fig. S3. Additionally, we also provided scatter plots (Fig. 3), funnel plots (Additional file 1: Fig. S4), and forest plots (Additional file 1: Fig. S2) to visually present the MR analysis. Building upon our sensitivity analysis results, we possess substantial confidence in the reliability and stability of the conclusions derived from the IVW method.

**Table 2:** Heterogeneity and pleiotropy analysis in forward MR analysis

Exposure	MR method	Cochran Q statistic	Egger intercept	Heterogeneity *P*_value	Pleiotropy *P*_value
AF	MR Egger	85.25	− 0.007	0.776	0.290
	IVW	86.38		0.771	
CAD	MR Egger	70.02	− 0.007	0.154	0.500
	IVW	70.57		0.165	
HF	MR Egger	16.17	− 0.020	0.024	0.713
	IVW	16.51		0.036	
MI	MR Egger	79.27	− 0.005	0.110	0.579
	IVW	79.65		0.121	
Stroke	MR Egger	13.53	− 0.028	0.486	0.591
	IVW	13.83		0.539	
IS	MR Egger	12.87	− 0.035	0.613	0.394
	IVW	13.64		0.626	

*MR* Mendelian randomization; *IVW* inverse variance weighted, *AF* atrial fibrillation; *CAD* coronary artery disease; *HF* heart failure; *MI* myocardial infarction; *IS* ischemic stroke

### Causal effects of FS on CVDs

**Fig. 4:**
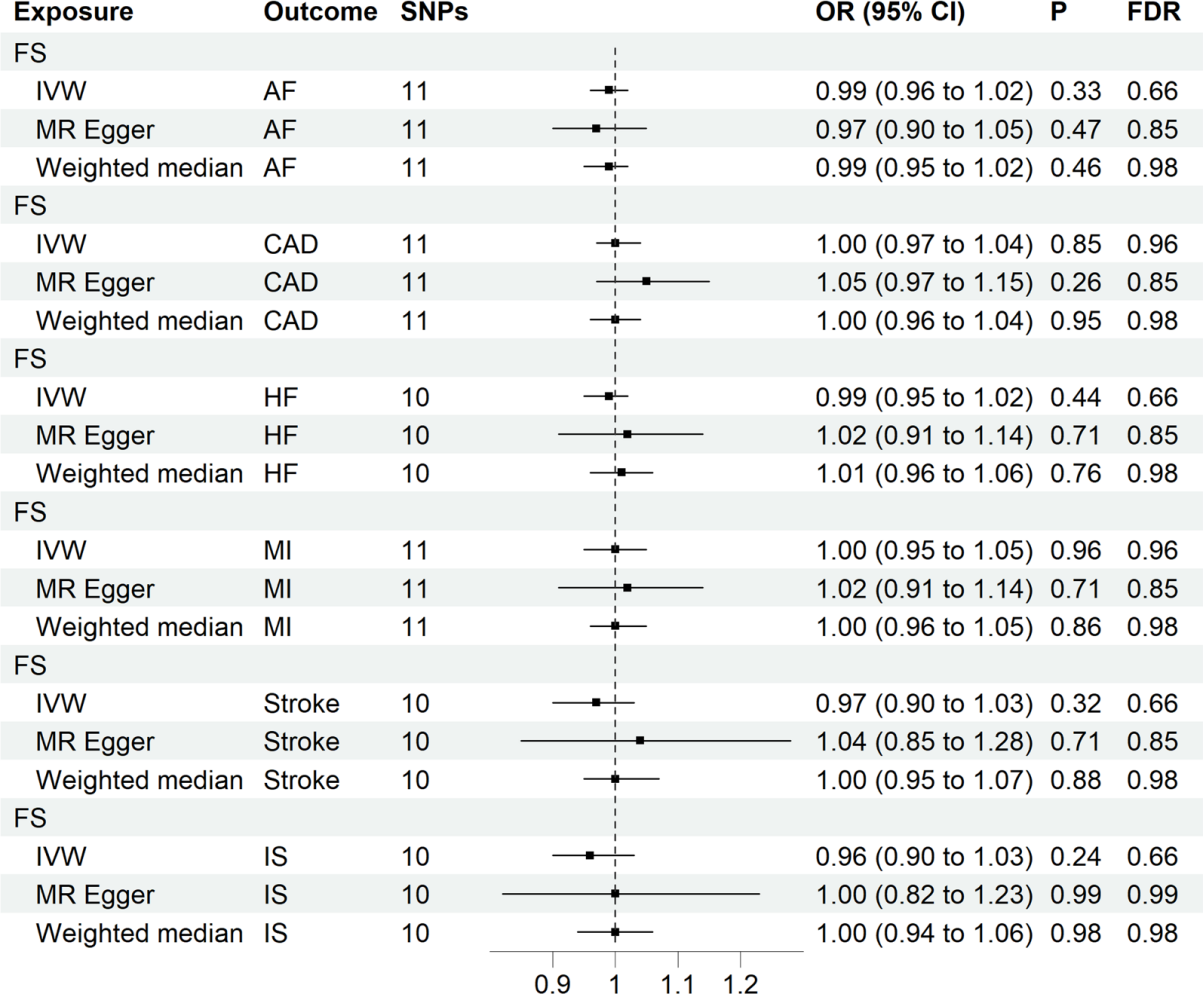
Forest plots of MR study using genetically predicted frozen shoulder with CVDs. IVW, inverse variance weighted; AF, atrial fibrillation; CAD, coronary artery disease; HF, heart failure; MI, myocardial infarction; IS, ischemic stroke. High risk: *P* < 0.05 and FDR < 0.05; potential risk: *P* < 0.05 and FDR > 0.05; unclear: *P* > 0.05; protective: *P* < 0.05 and FDR < 0.05

In the reverse MR analysis, we effectively screened and identified 12 SNPs associated with FS. It is worth noting that the SNP rs28599891 was excluded from the analysis due to its palindromic characteristics. Unfortunately, genetic data for SNP rs35464280 could not be obtained in the stroke, IS, and HF aspects. Consequently, in the final MR studies involving AF, CAD, HF, MI, stroke, IS, and FS, analyses were conducted with 11, 11, 10, 11, 10, and 10 SNPs, respectively (Additional file 1: Fig. S1). Nevertheless, our study did not uncover any causality between CVDs and FS (AF, OR [95% CI] = 0.99 (0.96 to 1.02), *P* = 0.33; CAD, OR [95% CI] = 1.00(0.97 to 1.04), *P* = 0.85; HF, OR [95% CI] = 0.99(0.95 to 1.02), *P* = 0.44; MI, OR [95% CI] = 1.00(0.95 to 1.05), *P* = 0.96; Stroke, OR [95% CI] = 0.97(0.90 to 1.03), *P* = 0.32; IS, OR [95% CI] = 0.96(0.90 to 1.03), *P* = 0.24), as indicated in Fig. 4. Encouragingly, these sensitivity analyses did not reveal any prominent anomalies, further reinforcing the reliability of our research conclusions (Additional file 1: Table S4). Additional detailed information, including characteristics of each SNP, can be found in Additional file 1: Table S3. To visually present our research findings more intuitively, Additional file 1: Figs. S5, S6, S7 and S8 depict the visual results of the relationship between FS and the 6 types of CVD.

## Discussion

We utilized a bidirectional two-sample MR study for the first time to investigate the causal relationships between CVDs and FS. The results indicated that some types of CVDs were related to the risk of FS. Specifically, genetic susceptibility to stroke and IS was significantly associated with FS risk, and this association remains significant even after FDR correction. Nevertheless, we lacked substantial evidence to establish a link between AF, CAD, HF, MI, and FS. However, we did not find sufficient evidence to support connections between AF, CAD, HF, MI, with FS. These findings exhibited robustness and consistency across various sensitivity analyses, further enhancing the reliability of our study results.

Previous studies have shown that stroke increases the risk of FS. This observation aligns with the conclusion of our study. Relevant studies indicated that the most common complication one year after stroke was shoulder pain, accounting for approximately 40% [30]. Most shoulder pain occurs within 2 months after onset, with 50% of them attributed to FS [31]. Over time, the risk of developing FS in participants 2 to 6 months after stroke occurrence increases four times [32]. Furthermore, MR studies have also verified a causality between IS and FS [14]. Firstly, stroke might trigger an inflammatory response in the shoulder joint, stimulate fibrosis of the muscles around the shoulder area, and subsequently elevate the risk of developing FS. Stroke results in cerebral ischemia or hemorrhage, causing the release of chemical compounds called inflammatory mediators when brain cells are damaged or die. These compounds include cytokines and inflammatory proteins, which then activate immune cells and inflammatory cells, initiating an inflammatory response [33, 34]. A broad consensus exists that the progression of FS is closely linked to inflammation and fibrosis. Studies have indicated a significant elevation of inflammatory cytokines such as interleukin (IL)-1α, IL-1β, tumor necrosis factor (TNF)-α, and cyclooxygenase-2 in the joint capsules of FS patients [35]. Extensive presence of inflammatory cells like T cells, B cells, macrophages, and mast cells has been widely detected in supraspinatus muscle interval samples from FS patients [36]. Secondly, central nervous system lesions following a stroke often lead to limb paralysis, progressively increasing the risk of joint effusion and muscle adhesions, ultimately contributing to the occurrence of FS [37, 38]. Finally, Some medications used in stroke treatment, especially those affecting blood coagulation and circulation, may have adverse effects on joints and muscles, thereby increasing the risk of developing FS. These medications may lead to blood vessel narrowing, affecting local blood supply and causing damage to joint tissues [39]. Additionally, drugs may modulate the immune response in joints through the immune system, leading to inflammation and damage. Side effects of medications may include discomfort or pain in muscles and joints, while a decrease in mobility may restrict normal shoulder joint movement, creating unfavorable conditions for the development of FS [40]. This complex interplay may make patients more prone to developing shoulder periarthritis.

It is noteworthy that there is currently no consensus on whether FS increases the risk of stroke. One study indicated that the risk of stroke in FS patients was 1.46-times (95% CI 1.32–1.62; *P* < 0.001) that of the control group, and even after adjusting for confounding factors, the risk remained elevated at 1.22-times(95% CI 1.06–1.40; *P* = 0.002) [8]. Subsequently, another follow-up study based on a large propensity score-matched population found no association between FS and an increased risk of stroke (OR [95% CI] = 0.93 (0.83–1.04), *P* = 0.178) [41]. This is consistent with our study findings. We mainly attribute this result to the substantial imbalance in demographic characteristics and distribution of vascular risk factors between the FS and non-FS groups in the study mentioned above [8]. Compared to the non-FS group, the FS group had a significantly older age, and the prevalence of diabetes and hyperlipidemia was 1.5 times higher. Despite adjustments in the multivariate regression analysis, the marked imbalances in these potential confounding variables could present challenges in effectively addressing these factors.

Interestingly, this study found no clear causal relationship between MI, CAD, and FS, which differs from previous study results and presents an intriguing trend. One study revealed that the risk of FS in CAD patients was 3.128-times (95% CI, 1.428–7.019; *P* = 0.005) that of non-FS patients [7]. On the one hand, shoulder pain in CAD patients may result in muscle contraction due to heart disease, thereby restricting shoulder joint movement. On the other hand, insufficient coronary artery blood supply and impaired cardiac function in CAD patients affect local vascular circulation, resulting in tissue ischemia and hypoxia, which can also cause shoulder pain [42]. Another follow-up study on the risk of FS patients indicated that over a 3-year follow-up period, the incidence risk of MI increased by 1.49-times (95% CI, 1.25–1.77; *P* < 0.001) [9]. They indicated that FS patients may exhibit elevated levels of C-reactive protein (CRP) and alterations in related immune complexes [43]. However, CRP plays a significant role in CVDs and is closely associated with their prognosis [44]. We speculate that the reasons for the inconsistency in study results may include the following two aspects: First, CAD, MI, and FS share some pathogenic factors, such as age, hyperlipidemia, and diabetes. CAD and MI may indirectly affect FS through these common pathogenic factors. MR studies may adopt more stringent methods, potentially better controlling confounding factors, while cross-sectional studies may yield inconsistent results due to the incomplete elimination of confounding factors. Second, MR studies focus more on the causal relationship's time sequence, while cross-sectional studies mainly reflect the correlation at the observed time points, potentially leading to differences in study outcomes. Importantly, the existing literature concerning this area of research is limited. Therefore, a cautious evaluation of these study results remains necessary, and future investigations should be conducted to attain a more comprehensive understanding of the interrelationships between MI, CAD, and FS.

However, our study results currently do not support a causal relationship between AF, HF, and FS, and no related research reports have been found to date. We speculate that AF and HF are CVD involving disruptions in heart structure and function, while FS is an inflammatory disorder around the joints. Since they impact distinct physiological systems and mechanisms, establishing a direct causal relationship is difficult.

We found that stroke increases the risk of developing FS, similar to Lv et al.'s study. However, we focus on the bidirectional causal relationship between six CVDs and FS, going beyond the singular relationship between stroke and FS. The results confirm that stroke increases the risk of FS, but there is no evidence that FS affects stroke. This deepens the understanding of the impact of FS on different CVDs. Although no causal relationship was found between FS and the other four CVDs, observational studies on the relationship between CAD, MI, and FS provided stronger evidence. Overall, by broadening the scope of our study, we ultimately confirmed the causal relationship between stroke and FS, providing a comprehensive and in-depth perspective on understanding the relationship between CVDs and FS.

The roles of various CVDs in the occurrence and progression of FS are complex and potentially interactive. Although our MR analysis was able to account for their interactive effects and assess their causality from a genetic perspective, our study has inherent limitations. Firstly, the results of this study are products of statistical analysis, and some correlations between CVDs and FS have not been reported, lacking theoretical support. Further, additional fundamental and clinical research is necessary to substantiate our observations. Secondly, our data were obtained from public databases that do not permit subgroup analyses for specific factors (such as age, gender, etc.). Future investigations into the correlation between CVDs and FS should account for the overall impact of these factors. Lastly, we exclusively utilized genetic data from individuals of European ancestry, limiting the generalizability of our study findings.

In summary, stroke is a high risk factor for FS. Orthopedic physicians should prioritize shoulder care for stroke patients, promptly assessing potential restrictions in shoulder range of motion to mitigate FS risk. However, further research is needed to validate and extend these findings.

### Supplementary Information

**Additional file 1. Table S1.** Characteristics of SNPs associated with cardiovascular disease. **Table S2.** Characteristics of SNPs associated with frozen shoulder. **Table S3.** Heterogeneity and pleiotropy analysis in reverse MR analysis. **Fig S1.** The forest plots for causal effect of cardiovascular disease on frozen shoulder. **Fig S2.** Leave-one-out sensitivity analysis for causal effect of cardiovascular disease on frozen shoulder. **Fig S3.** The funnel chart for causal effect of cardiovascular disease on frozen shoulder. **Fig. S4.** The scatter plots for causal effect of frozen shoulder on cardiovascular disease. **Fig. S5.** The forest plots for causal effect of frozen shoulder on cardiovascular disease. **Fig. S6.** Leave-one-out sensitivity analysis for causal effect of frozen shoulder on cardiovascular disease. **Fig. S7.** The funnel chart for causal effect of frozen shoulder on cardiovascular disease.

## Data Availability

All the Mendelian randomization study files are available from GWAS. (URL https://gwas.mrcieu.ac.uk).

## Abbreviations

CVD: Cardiovascular disease
FS: Frozen shoulder
MR: Mendelian randomized
RCTs: Randomized controlled trials
AF: Atrial fibrillation
CAD: Coronary artery disease
HF: Heart failure
MI: Myocardial infarction
IS: Ischemic stroke
GWAS: Genome-wide association studies
IVW: Inverse variance weighting
WM: Weighted median
IV: Instrumental variable
FDR: False discovery rate
MAF: Minimum allele frequency
SNP: Single-nucleotide polymorphism
OR: Odds ratio
CI: Confidence interval
*β*: Regression coefficient
SE: Standard error
