# Supplementary material for: Bidirectional two-sample Mendelian randomization analysis identifies causal associations between cardiovascular diseases and frozen shoulder
Source: J Orthop Surg Res. 2024 Feb 3;19:116. doi: 10.1186/s13018-024-04600-7 (PMC10837867; doi:10.1186/s13018-024-04600-7)
Supplement: Supplementary file 1 — Additional file 1. Table S1. Characteristics of SNPs associated with cardiovascular disease. Table S2. Characteristics of SNPs associated with frozen shoulder. Table S3. Heterogeneity and pleiotropy analysis in reverse MR analysis. Fig S1. The forest plots for causal effect of cardiovascular disease on frozen shoulder. Fig S2. Leave-one-out sensitivity analysis for causal effect of cardiovascular disease on frozen shoulder. Fig S3. The funnel chart for causal effect of cardiovascular disease on frozen shoulder. Fig. S4. The scatter plots for causal effect of frozen shoulder on cardiovascular disease. Fig. S5. The forest plots for causal effect of frozen shoulder on cardiovascular disease. Fig. S6. Leave-one-out sensitivity analysis for causal effect of frozen shoulder on cardiovascular disease. Fig. S7. The funnel chart for causal effect of frozen shoulder on cardiovascular disease. [file 13018_2024_4600_MOESM1_ESM.docx]

***Supplementary Materials***

**SUPPLEMENTARY NOTES**

**Supplementary Table 1** | Summary of Mendelian randomization method.

**Supplementary Table 2** | Characteristics of SNPs associated with cardiovascular disease.

**Supplementary Table 3**| Characteristics of SNPs associated with frozen shoulder.

**Supplementary Table 4** | Heterogeneity and pleiotropy analysis in reverse MR analysis.

**Supplementary Figure 1 |** SNP screening flow chart.

**Supplementary Figure 2 |** The forest plots for causal effect of cardiovascular disease on frozen shoulder.

**Supplementary Figure 3 |** Leave-one-out sensitivity analysis for causal effect of cardiovascular disease on frozen shoulder.

**Supplementary Figure 4 |** The funnel chart for causal effect of cardiovascular disease on frozen shoulder.

**Supplementary Figure 5 |** The scatter plots for causal effect of frozen shoulder on cardiovascular disease.

**Supplementary Figure 6 |** The forest plots for causal effect of frozen shoulder on cardiovascular disease.

**Supplementary Figure 7 |** Leave-one-out sensitivity analysis for causal effect of frozen shoulder on cardiovascular disease.

**Supplementary Figure 8 |** The funnel chart for causal effect of frozen shoulder on cardiovascular disease.

**Supplementary Table 1** | Summary of Mendelian randomization method.

| **MR method** | **Strengths and weaknesses** |
| --- | --- |
| IVW | A weighted mean of individual variant effects on the outcome, which provides an estimate equivalent to MR using individual‐level data, assuming the genetic variants are uncorrelated. The inverse-variance weighted method has optimal statistical power, but assumes all variants are valid instruments. Estimates are biased if there is directional pleiotropy (when the average value of the pleiotropy distribution is non-zero) |
| MR-Egger | Quantifies directional pleiotropy and accounts for it to provide an unbiased estimate even if all SNPs have pleiotropic effects. It requires the size of pleiotropic effects to be independent of the size of the variants’ effects on the exposure (the InSIDE assumption), which is not verifiable. It is sensitive to outliers and less efficient (results in wide CIs) |
| Weighted median | Robust to outliers; it provides unbiased estimate when up to half of the SNPs violate the instrumental variable assumptions, but may be less efficient |

**Supplementary Table 2** | Characteristics of SNPs associated with cardiovascular disease.

| **AF** |  |  |  |  |  |  |  |  |  |
| --- | --- | --- | --- | --- | --- | --- | --- | --- | --- |
| **SNP** | **EA** | **Position** | **EAF** | **BETA** | **SE** | **P** | **N** | **R2** | **F** |
| rs10141892 | C | 35184323 | 0.58 | -0.045 | 0.007 | 2.95E-11 | 1030836 | 9.93E-04 | 1025 |
| rs10213171a | G | 148937537 | 0.06 | 0.091 | 0.013 | 1.32E-11 | 1030836 | 9.47E-04 | 977 |
| rs10458662 | G | 77936670 | 0.17 | 0.054 | 0.009 | 6.93E-10 | 1030836 | 8.44E-04 | 870 |
| rs10520002 | A | 127819132 | 0.10 | 0.063 | 0.011 | 2.85E-08 | 1030836 | 6.98E-04 | 720 |
| rs10520260 | G | 174447349 | 0.32 | -0.046 | 0.007 | 3.36E-10 | 1030836 | 9.11E-04 | 940 |
| rs10753933 | G | 203026214 | 0.55 | -0.061 | 0.007 | 9.84E-20 | 1030836 | 1.83E-03 | 1895 |
| rs10773657 | A | 123327900 | 0.86 | -0.058 | 0.010 | 2.54E-08 | 1030836 | 7.87E-04 | 811 |
| rs10804493 | A | 111554426 | 0.65 | 0.056 | 0.007 | 1.63E-15 | 1030836 | 1.42E-03 | 1461 |
| rs10821415 | A | 97713459 | 0.41 | 0.082 | 0.007 | 2.92E-34 | 1030836 | 3.27E-03 | 3380 |
| rs10842383 | T | 24771967 | 0.15 | -0.099 | 0.010 | 2.88E-25 | 1030836 | 2.46E-03 | 2541 |
| rs11191116 | T | 103555611 | 0.35 | -0.041 | 0.007 | 4.42E-09 | 1030836 | 7.63E-04 | 787 |
| rs11264280 | T | 154862952 | 0.33 | 0.135 | 0.007 | 3.07E-79 | 1030836 | 8.06E-03 | 8376 |
| rs11598047 | G | 105342672 | 0.16 | 0.154 | 0.009 | 8.95E-66 | 1030836 | 6.42E-03 | 6658 |
| rs11773845 | A | 116191301 | 0.59 | 0.105 | 0.007 | 2.39E-55 | 1030836 | 5.39E-03 | 5588 |
| rs117984853 | T | 149399100 | 0.10 | 0.123 | 0.012 | 1.34E-24 | 1030836 | 2.75E-03 | 2838 |
| rs12245149 | A | 65321147 | 0.47 | -0.047 | 0.007 | 1.66E-12 | 1030836 | 1.10E-03 | 1137 |
| rs12426679 | T | 76237987 | 0.53 | -0.039 | 0.007 | 4.95E-09 | 1030836 | 7.62E-04 | 786 |
| rs1278493 | A | 135814009 | 0.56 | -0.039 | 0.007 | 8.77E-09 | 1030836 | 7.44E-04 | 768 |
| rs133885 | A | 26159289 | 0.44 | 0.041 | 0.007 | 2.22E-09 | 1030836 | 8.07E-04 | 833 |
| rs140185678 | A | 2003016 | 0.04 | 0.166 | 0.022 | 2.43E-14 | 1030836 | 1.86E-03 | 1925 |
| rs1458038 | T | 81164723 | 0.31 | 0.043 | 0.007 | 1.74E-09 | 1030836 | 8.04E-04 | 829 |
| rs146518726 | A | 51535039 | 0.03 | 0.161 | 0.021 | 8.27E-15 | 1030836 | 1.63E-03 | 1688 |
| rs1563304 | T | 44874453 | 0.18 | 0.064 | 0.009 | 2.56E-12 | 1030836 | 1.21E-03 | 1253 |
| rs17171711 | T | 137364795 | 0.18 | 0.109 | 0.009 | 1.95E-35 | 1030836 | 3.44E-03 | 3562 |
| rs17380837 | T | 26345526 | 0.31 | -0.050 | 0.007 | 4.80E-12 | 1030836 | 1.07E-03 | 1102 |
| rs1838747 | G | 114426668 | 0.50 | 0.039 | 0.007 | 4.13E-09 | 1030836 | 7.64E-04 | 789 |
| rs1906615 | T | 111701798 | 0.20 | 0.366 | 0.008 | 3.26E-100 | 1030836 | 4.27E-02 | 45951 |
| rs2031522 | G | 87821501 | 0.38 | -0.044 | 0.007 | 1.47E-10 | 1030836 | 8.92E-04 | 921 |
| rs2274115 | G | 139094773 | 0.70 | 0.049 | 0.008 | 1.69E-10 | 1030836 | 9.96E-04 | 1027 |
| rs2288327 | G | 179411665 | 0.16 | 0.092 | 0.009 | 7.26E-25 | 1030836 | 2.23E-03 | 2302 |
| rs2359171a | A | 73053022 | 0.18 | 0.175 | 0.009 | 4.65E-91 | 1030836 | 8.84E-03 | 9196 |
| rs2540949a | T | 65284231 | 0.38 | -0.066 | 0.007 | 2.95E-22 | 1030836 | 2.06E-03 | 2124 |
| rs2738413 | G | 64679960 | 0.50 | -0.078 | 0.007 | 2.55E-31 | 1030836 | 3.03E-03 | 3129 |
| rs2739197a | G | 111543323 | 0.25 | 0.116 | 0.009 | 3.20E-41 | 1030836 | 4.99E-03 | 5169 |
| rs2739197a | G | 111543323 | 0.25 | 0.116 | 0.009 | 3.20E-41 | 1030836 | 4.99E-03 | 5169 |
| rs2759301 | A | 80994288 | 0.45 | 0.039 | 0.007 | 5.04E-09 | 1030836 | 7.54E-04 | 778 |
| rs2834618 | G | 36119111 | 0.11 | -0.094 | 0.011 | 3.41E-17 | 1030836 | 1.68E-03 | 1738 |
| rs28387148 | T | 127433465 | 0.11 | 0.074 | 0.011 | 6.25E-11 | 1030836 | 1.03E-03 | 1066 |
| rs284277 | A | 10790797 | 0.62 | -0.042 | 0.007 | 1.25E-09 | 1030836 | 8.41E-04 | 868 |
| rs2860482 | C | 57105938 | 0.73 | -0.054 | 0.008 | 1.21E-12 | 1030836 | 1.16E-03 | 1197 |
| rs28631169 | T | 23888183 | 0.20 | 0.052 | 0.008 | 5.35E-10 | 1030836 | 8.66E-04 | 894 |
| rs2885697 | T | 41544279 | 0.65 | -0.044 | 0.007 | 2.88E-10 | 1030836 | 8.79E-04 | 907 |
| rs3176326 | A | 36647289 | 0.20 | -0.063 | 0.009 | 1.42E-13 | 1030836 | 1.25E-03 | 1286 |
| rs337705 | G | 113737062 | 0.37 | 0.056 | 0.007 | 1.63E-16 | 1030836 | 1.49E-03 | 1539 |
| rs34080181 | A | 66454191 | 0.38 | -0.045 | 0.007 | 1.28E-10 | 1030836 | 9.36E-04 | 966 |
| rs34936990 | A | 105523416 | 0.12 | 0.129 | 0.010 | 2.95E-37 | 1030836 | 3.55E-03 | 3677 |
| rs34969716 | A | 18210109 | 0.31 | 0.070 | 0.008 | 1.60E-19 | 1030836 | 2.09E-03 | 2159 |
| rs35544454a | T | 213266003 | 0.19 | -0.059 | 0.009 | 1.10E-11 | 1030836 | 1.08E-03 | 1110 |
| rs35569628 | C | 113872712 | 0.22 | -0.045 | 0.008 | 1.38E-08 | 1030836 | 7.08E-04 | 730 |
| rs35963991 | T | 11495702 | 0.15 | 0.053 | 0.010 | 2.80E-08 | 1030836 | 7.01E-04 | 723 |
| rs3943207 | T | 21845619 | 0.12 | -0.064 | 0.010 | 6.92E-10 | 1030836 | 8.39E-04 | 866 |
| rs4073778 | A | 116297758 | 0.56 | 0.049 | 0.007 | 4.96E-13 | 1030836 | 1.16E-03 | 1199 |
| rs4252627 | T | 37868715 | 0.67 | -0.042 | 0.007 | 5.63E-09 | 1030836 | 7.64E-04 | 788 |
| rs4587869a | C | 32992334 | 0.28 | 0.072 | 0.008 | 1.19E-20 | 1030836 | 2.09E-03 | 2158 |
| rs4642101 | G | 12843862 | 0.64 | 0.071 | 0.007 | 2.95E-24 | 1030836 | 2.30E-03 | 2374 |
| rs464901 | C | 18597502 | 0.34 | -0.051 | 0.007 | 1.53E-12 | 1030836 | 1.15E-03 | 1187 |
| rs4757877 | G | 20010291 | 0.76 | -0.072 | 0.008 | 2.93E-20 | 1030836 | 1.93E-03 | 1996 |
| rs4935786a | A | 121661507 | 0.73 | -0.046 | 0.008 | 4.85E-09 | 1030836 | 8.40E-04 | 866 |
| rs4946333 | G | 118565665 | 0.49 | 0.064 | 0.007 | 5.47E-22 | 1030836 | 2.04E-03 | 2108 |
| rs4965430a | G | 99268850 | 0.61 | -0.044 | 0.007 | 1.26E-10 | 1030836 | 9.22E-04 | 952 |
| rs55734480 | A | 14372009 | 0.25 | 0.055 | 0.008 | 2.20E-12 | 1030836 | 1.12E-03 | 1160 |
| rs55985730 | G | 128417044 | 0.06 | 0.087 | 0.015 | 5.24E-09 | 1030836 | 8.48E-04 | 875 |
| rs56201652 | A | 92278116 | 0.27 | -0.053 | 0.008 | 1.74E-12 | 1030836 | 1.10E-03 | 1139 |
| rs56326533 | C | 201168758 | 0.39 | 0.069 | 0.007 | 6.28E-24 | 1030836 | 2.24E-03 | 2311 |
| rs577676 | T | 170587340 | 0.44 | -0.092 | 0.007 | 1.62E-43 | 1030836 | 4.19E-03 | 4342 |
| rs60902112 | T | 194800853 | 0.23 | 0.045 | 0.008 | 1.72E-08 | 1030836 | 6.93E-04 | 715 |
| rs62254082 | C | 69417585 | 0.39 | 0.040 | 0.007 | 6.34E-09 | 1030836 | 7.74E-04 | 799 |
| rs62377206 | A | 168383543 | 0.06 | 0.085 | 0.015 | 8.21E-09 | 1030836 | 7.49E-04 | 773 |
| rs62521286 | G | 124551975 | 0.07 | 0.120 | 0.014 | 4.50E-19 | 1030836 | 1.79E-03 | 1847 |
| rs6462079 | A | 28415827 | 0.72 | 0.047 | 0.008 | 8.79E-10 | 1030836 | 8.74E-04 | 902 |
| rs6546620 | C | 26159940 | 0.80 | 0.060 | 0.009 | 3.19E-12 | 1030836 | 1.16E-03 | 1197 |
| rs6560886 | C | 133150210 | 0.79 | 0.051 | 0.009 | 1.49E-08 | 1030836 | 8.68E-04 | 895 |
| rs6580277 | G | 142818123 | 0.24 | 0.067 | 0.008 | 1.64E-17 | 1030836 | 1.62E-03 | 1676 |
| rs6596717 | A | 106427609 | 0.60 | -0.040 | 0.007 | 3.00E-09 | 1030836 | 7.80E-04 | 805 |
| rs6665642 | T | 154802139 | 0.12 | -0.062 | 0.011 | 3.06E-08 | 1030836 | 7.98E-04 | 823 |
| rs6689306 | G | 154395946 | 0.59 | -0.046 | 0.007 | 1.36E-11 | 1030836 | 1.03E-03 | 1059 |
| rs6747542 | C | 70106832 | 0.46 | -0.055 | 0.007 | 1.10E-16 | 1030836 | 1.53E-03 | 1576 |
| rs6771054 | C | 89489529 | 0.40 | -0.046 | 0.007 | 2.42E-11 | 1030836 | 1.01E-03 | 1037 |
| rs6790396a | G | 38771925 | 0.60 | 0.063 | 0.007 | 2.40E-20 | 1030836 | 1.89E-03 | 1955 |
| rs67969609a | G | 145760353 | 0.07 | 0.071 | 0.013 | 1.71E-08 | 1030836 | 6.67E-04 | 688 |
| rs6838973 | T | 111765495 | 0.44 | -0.151 | 0.007 | 1.03E-111 | 1030836 | 1.13E-02 | 11781 |
| rs6882776 | A | 172664163 | 0.28 | -0.071 | 0.007 | 9.64E-22 | 1030836 | 2.05E-03 | 2121 |
| rs6994744 | C | 141740868 | 0.50 | 0.041 | 0.007 | 1.09E-09 | 1030836 | 8.20E-04 | 846 |
| rs71454237 | A | 70013415 | 0.21 | -0.062 | 0.008 | 1.78E-13 | 1030836 | 1.27E-03 | 1312 |
| rs7172038 | G | 73667255 | 0.16 | 0.112 | 0.009 | 4.78E-36 | 1030836 | 3.37E-03 | 3482 |
| rs7224711 | T | 76772288 | 0.52 | -0.037 | 0.007 | 3.72E-08 | 1030836 | 6.65E-04 | 686 |
| rs7225165 | A | 1309850 | 0.11 | -0.066 | 0.011 | 3.20E-09 | 1030836 | 8.62E-04 | 889 |
| rs72694603 | T | 112458893 | 0.31 | -0.055 | 0.007 | 2.26E-14 | 1030836 | 1.32E-03 | 1362 |
| rs72700114a | C | 170193825 | 0.08 | 0.202 | 0.013 | 3.29E-54 | 1030836 | 5.71E-03 | 5919 |
| rs72811294a | C | 12618680 | 0.11 | -0.072 | 0.011 | 9.67E-12 | 1030836 | 1.04E-03 | 1073 |
| rs72926475 | A | 86594487 | 0.12 | -0.068 | 0.010 | 2.37E-11 | 1030836 | 1.01E-03 | 1037 |
| rs72966339 | T | 122398241 | 0.37 | -0.062 | 0.007 | 7.42E-19 | 1030836 | 1.76E-03 | 1822 |
| rs73041705 | C | 24463235 | 0.30 | -0.044 | 0.007 | 1.55E-09 | 1030836 | 8.22E-04 | 848 |
| rs73366713 | A | 16415751 | 0.14 | -0.104 | 0.010 | 1.53E-25 | 1030836 | 2.57E-03 | 2660 |
| rs74500426 | T | 174642789 | 0.08 | -0.092 | 0.013 | 4.29E-13 | 1030836 | 1.20E-03 | 1235 |
| rs74832855 | G | 170171598 | 0.04 | 0.122 | 0.018 | 1.43E-11 | 1030836 | 1.05E-03 | 1085 |
| rs74884082 | T | 73249419 | 0.25 | -0.049 | 0.008 | 3.48E-10 | 1030836 | 9.10E-04 | 939 |
| rs7508 | A | 17913970 | 0.71 | 0.071 | 0.008 | 1.69E-21 | 1030836 | 2.08E-03 | 2146 |
| rs7529220 | C | 22282619 | 0.85 | 0.062 | 0.010 | 1.98E-10 | 1030836 | 1.00E-03 | 1032 |
| rs7574892 | A | 175512820 | 0.48 | 0.055 | 0.007 | 1.98E-16 | 1030836 | 1.52E-03 | 1571 |
| rs76097649 | A | 128764570 | 0.09 | 0.115 | 0.012 | 1.26E-20 | 1030836 | 2.24E-03 | 2316 |
| rs7612445 | T | 179172979 | 0.19 | 0.049 | 0.008 | 4.81E-09 | 1030836 | 7.42E-04 | 765 |
| rs77316573 | T | 2265271 | 0.20 | 0.053 | 0.009 | 3.26E-09 | 1030836 | 8.92E-04 | 921 |
| rs775498 | G | 70071513 | 0.28 | 0.042 | 0.007 | 1.05E-08 | 1030836 | 7.21E-04 | 744 |
| rs7789146 | A | 150661409 | 0.18 | -0.058 | 0.009 | 2.12E-11 | 1030836 | 1.00E-03 | 1033 |
| rs7915134 | T | 75420180 | 0.14 | -0.117 | 0.010 | 1.42E-34 | 1030836 | 3.36E-03 | 3477 |
| rs79187193 | A | 147255831 | 0.06 | -0.116 | 0.015 | 3.15E-14 | 1030836 | 1.45E-03 | 1496 |
| rs8088085 | C | 48708548 | 0.46 | -0.037 | 0.007 | 4.79E-08 | 1030836 | 6.63E-04 | 684 |
| rs883079 | T | 114793240 | 0.71 | 0.098 | 0.007 | 2.84E-40 | 1030836 | 3.98E-03 | 4123 |
| rs9506925 | T | 23368943 | 0.27 | 0.045 | 0.008 | 2.72E-09 | 1030836 | 7.89E-04 | 814 |
| rs9953366 | C | 46474192 | 0.66 | 0.049 | 0.007 | 1.82E-11 | 1030836 | 1.07E-03 | 1107 |
| **CAD** |  |  |  |  |  |  |  |  |  |
| **SNP** | **EA** | **Position** | **EAF** | **BETA** | **SE** | **P** | **N** | **R2** | **F** |
| rs10131519 | C | 100359294 | NA | 0.050 | 0.009 | 2.6E-08 | 547261 | 5.66E-05 | 31 |
| rs10793514 | C | 44496971 | NA | 0.051 | 0.007 | 4.4E-12 | 547261 | 8.76E-05 | 48 |
| rs10841443a | G | 20220033 | NA | 0.043 | 0.007 | 5.2E-09 | 547261 | 6.24E-05 | 34 |
| rs10857147a | T | 81181072 | NA | 0.046 | 0.008 | 2.3E-09 | 547261 | 6.52E-05 | 36 |
| rs11057840 | C | 125316055 | NA | 0.071 | 0.010 | 1.4E-12 | 547261 | 9.16E-05 | 50 |
| rs11072783 | A | 78965966 | NA | -0.056 | 0.009 | 1.2E-09 | 547261 | 6.74E-05 | 37 |
| rs112043140 | T | 46585022 | NA | 0.046 | 0.008 | 2.9E-08 | 547261 | 5.63E-05 | 31 |
| rs1148497 | G | 102856517 | NA | -0.046 | 0.008 | 4.7E-08 | 547261 | 5.45E-05 | 30 |
| rs11591147 | T | 55505647 | NA | -0.237 | 0.027 | 1.5E-18 | 547261 | 1.41E-04 | 77 |
| rs11601507 | A | 5701074 | NA | 0.081 | 0.014 | 2.1E-09 | 547261 | 6.56E-05 | 36 |
| rs11617955a | A | 110818102 | NA | -0.085 | 0.011 | 4E-14 | 547261 | 1.04E-04 | 57 |
| rs11619113a | G | 110918660 | NA | 0.058 | 0.010 | 3E-08 | 547261 | 5.61E-05 | 31 |
| rs11637783 | C | 79139000 | NA | -0.064 | 0.007 | 9.7E-20 | 547261 | 1.51E-04 | 83 |
| rs11673093 | A | 45742094 | NA | 0.044 | 0.008 | 3E-08 | 547261 | 5.61E-05 | 31 |
| rs117733303 | G | 160922870 | NA | 0.452 | 0.026 | 8.4E-66 | 547261 | 5.36E-04 | 294 |
| rs12212146 | C | 161125454 | NA | -0.089 | 0.014 | 2.1E-10 | 547261 | 7.38E-05 | 40 |
| rs12315434 | C | 57780936 | NA | -0.050 | 0.008 | 2.8E-09 | 547261 | 6.45E-05 | 35 |
| rs1250259a | A | 216300482 | NA | -0.047 | 0.008 | 2.9E-09 | 547261 | 6.44E-05 | 35 |
| rs12740374 | T | 109817590 | NA | -0.104 | 0.008 | 8.2E-36 | 547261 | 2.85E-04 | 156 |
| rs12930452 | G | 75462055 | NA | 0.049 | 0.007 | 5.5E-12 | 547261 | 8.68E-05 | 47 |
| rs1412444 | T | 91002927 | NA | 0.049 | 0.007 | 1.4E-11 | 547261 | 8.34E-05 | 46 |
| rs146534110 | T | 160578069 | NA | 0.209 | 0.031 | 2.2E-11 | 547261 | 8.18E-05 | 45 |
| rs15285 | T | 19824667 | NA | -0.048 | 0.008 | 4E-10 | 547261 | 7.15E-05 | 39 |
| rs16986953 | A | 19942473 | NA | 0.081 | 0.013 | 1.4E-09 | 547261 | 6.69E-05 | 37 |
| rs17114046 | G | 56966350 | NA | -0.097 | 0.012 | 2.8E-16 | 547261 | 1.22E-04 | 67 |
| rs17228058 | G | 67450305 | NA | -0.052 | 0.008 | 4E-10 | 547261 | 7.15E-05 | 39 |
| rs17263917 | A | 9552338 | NA | -0.062 | 0.010 | 3.2E-10 | 547261 | 7.22E-05 | 40 |
| rs17465982 | A | 222837939 | NA | 0.066 | 0.008 | 7.3E-18 | 547261 | 1.35E-04 | 74 |
| rs17612693a | A | 148365339 | NA | 0.081 | 0.010 | 3.2E-16 | 547261 | 1.22E-04 | 67 |
| rs1966248a | T | 134159622 | NA | -0.071 | 0.008 | 1.5E-20 | 547261 | 1.58E-04 | 86 |
| rs2107595 | A | 19049388 | NA | 0.077 | 0.010 | 9.4E-16 | 547261 | 1.18E-04 | 65 |
| rs2128739 | C | 103673277 | NA | -0.066 | 0.008 | 1.0E-17 | 547261 | 1.34E-04 | 73 |
| rs2140480 | C | 110526109 | NA | -0.044 | 0.008 | 2.2E-08 | 547261 | 5.72E-05 | 31 |
| rs2166529 | T | 85742175 | NA | 0.056 | 0.007 | 1.1E-15 | 547261 | 1.17E-04 | 64 |
| rs223290 | C | 57106712 | NA | -0.097 | 0.014 | 6.7E-12 | 547261 | 8.61E-05 | 47 |
| rs2293251 | G | 138124114 | NA | 0.061 | 0.010 | 3.4E-10 | 547261 | 7.21E-05 | 39 |
| rs246600 | T | 142516897 | NA | 0.044 | 0.007 | 4.0E-10 | 547261 | 7.15E-05 | 39 |
| rs2649999 | C | 121380544 | NA | -0.043 | 0.008 | 1.6E-08 | 547261 | 5.83E-05 | 32 |
| rs28451064 | A | 35593827 | NA | 0.099 | 0.011 | 2.7E-20 | 547261 | 1.56E-04 | 85 |
| rs28601761a | G | 126500031 | NA | -0.057 | 0.007 | 2.8E-15 | 547261 | 1.14E-04 | 62 |
| rs2891168 | G | 22098619 | NA | 0.170 | 0.007 | 1.8E-131 | 547261 | 1.09E-03 | 595 |
| rs3127580 | T | 160710851 | NA | 0.085 | 0.010 | 2.6E-18 | 547261 | 1.39E-04 | 76 |
| rs318719 | C | 11496981 | NA | 0.075 | 0.013 | 5.6E-09 | 547261 | 6.21E-05 | 34 |
| rs3741380 | A | 65349063 | NA | 0.041 | 0.007 | 3.6E-09 | 547261 | 6.36E-05 | 35 |
| rs3796581 | G | 156642884 | NA | -0.069 | 0.009 | 2.6E-14 | 547261 | 1.06E-04 | 58 |
| rs3918226 | T | 150690176 | NA | 0.097 | 0.013 | 2.3E-13 | 547261 | 9.81E-05 | 54 |
| rs4280376 | C | 17861209 | NA | 0.056 | 0.009 | 5.8E-10 | 547261 | 7.01E-05 | 38 |
| rs4299376 | T | 44072576 | NA | -0.045 | 0.008 | 1.5E-09 | 547261 | 6.67E-05 | 37 |
| rs4678145a | C | 124450081 | NA | 0.064 | 0.010 | 6.9E-10 | 547261 | 6.95E-05 | 38 |
| rs4762479 | T | 95495041 | NA | -0.085 | 0.013 | 2.5E-10 | 547261 | 7.31E-05 | 40 |
| rs4766578a | A | 111904371 | NA | -0.065 | 0.007 | 5.3E-20 | 547261 | 1.53E-04 | 84 |
| rs4803455 | A | 41851509 | NA | -0.050 | 0.007 | 1.1E-12 | 547261 | 9.25E-05 | 51 |
| rs4932373 | C | 91429287 | NA | 0.069 | 0.007 | 3.3E-20 | 547261 | 1.55E-04 | 85 |
| rs55730499 | T | 161005610 | NA | 0.311 | 0.013 | 6.3E-120 | 547261 | 9.90E-04 | 542 |
| rs56210800a | G | 124472592 | NA | 0.063 | 0.010 | 1.8E-09 | 547261 | 6.61E-05 | 36 |
| rs566818 | G | 75158350 | NA | -0.042 | 0.008 | 3.2E-08 | 547261 | 5.59E-05 | 31 |
| rs604723 | C | 100610546 | NA | 0.050 | 0.008 | 2.8E-10 | 547261 | 7.27E-05 | 40 |
| rs6460942 | C | 12420989 | NA | -0.059 | 0.010 | 7.6E-09 | 547261 | 6.10E-05 | 33 |
| rs6511720 | T | 11202306 | NA | -0.108 | 0.011 | 3.9E-24 | 547261 | 1.88E-04 | 103 |
| rs655720 | T | 136103267 | NA | 0.054 | 0.008 | 2.9E-12 | 547261 | 8.91E-05 | 49 |
| rs685031 | A | 31881731 | NA | 0.046 | 0.007 | 3E-10 | 547261 | 7.25E-05 | 40 |
| rs6905288 | A | 43758873 | NA | 0.041 | 0.007 | 4.2E-09 | 547261 | 6.31E-05 | 35 |
| rs701145 | C | 154054799 | NA | 0.066 | 0.010 | 2.4E-11 | 547261 | 8.15E-05 | 45 |
| rs7209460 | T | 2048713 | NA | 0.052 | 0.008 | 7.6E-12 | 547261 | 8.56E-05 | 47 |
| rs72934535 | C | 203968973 | NA | 0.100 | 0.012 | 3.7E-18 | 547261 | 1.38E-04 | 75 |
| rs7412 | T | 45412079 | NA | -0.137 | 0.013 | 7.3E-27 | 547261 | 2.10E-04 | 115 |
| rs7502499 | A | 47490102 | NA | 0.053 | 0.008 | 3.3E-12 | 547261 | 8.86E-05 | 48 |
| rs77347777 | T | 52848207 | NA | -0.061 | 0.011 | 3.7E-08 | 547261 | 5.54E-05 | 30 |
| rs7896600a | C | 12255175 | NA | 0.045 | 0.008 | 2.3E-08 | 547261 | 5.70E-05 | 31 |
| rs8003602 | C | 100148961 | NA | 0.055 | 0.008 | 4.1E-12 | 547261 | 8.78E-05 | 48 |
| rs9337951 | A | 30317073 | NA | 0.050 | 0.008 | 4.6E-11 | 547261 | 7.92E-05 | 43 |
| rs9349379 | G | 12903957 | NA | 0.093 | 0.007 | 6.9E-39 | 547261 | 3.11E-04 | 170 |
| rs9554448 | C | 98859722 | NA | -0.063 | 0.011 | 3.9E-08 | 547261 | 5.52E-05 | 30 |
| rs964184a | C | 116648917 | NA | -0.060 | 0.010 | 2.2E-09 | 547261 | 6.53E-05 | 36 |
| **HF** |  |  |  |  |  |  |  |  |  |
| **SNP** | **EA** | **Position** | **EAF** | **BETA** | **SE** | **P** | **N** | **R2** | **F** |
| rs11745324 | A | 137012171 | NA | -0.053 | 0.010 | 2.34E-08 | 977323 | 3.16E-05 | 31 |
| rs1510226 | C | 160816409 | NA | 0.162 | 0.029 | 1.27E-08 | 977323 | 3.31E-05 | 32 |
| rs1556516a | C | 22100176 | NA | 0.062 | 0.008 | 1.57E-15 | 977323 | 6.51E-05 | 64 |
| rs17042102 | A | 111668626 | NA | 0.110 | 0.012 | 5.71E-20 | 977323 | 8.50E-05 | 83 |
| rs17617337 | T | 121426884 | NA | -0.056 | 0.010 | 3.65E-09 | 977323 | 3.57E-05 | 35 |
| rs4135240 | C | 36647680 | NA | -0.049 | 0.008 | 6.84E-09 | 977323 | 3.43E-05 | 33 |
| rs4746140a | C | 75417249 | NA | -0.067 | 0.011 | 1.1E-09 | 977323 | 3.82E-05 | 37 |
| rs4766578a | A | 111904371 | NA | -0.043 | 0.008 | 4.9E-08 | 977323 | 3.07E-05 | 30 |
| rs55730499 | T | 161005610 | NA | 0.106 | 0.016 | 1.83E-11 | 977323 | 4.65E-05 | 45 |
| rs56094641 | G | 53806453 | NA | 0.045 | 0.008 | 1.21E-08 | 977323 | 3.30E-05 | 32 |
| rs600038 | C | 136151806 | NA | 0.057 | 0.010 | 3.68E-09 | 977323 | 3.59E-05 | 35 |
| rs660240 | C | 109817838 | NA | 0.061 | 0.010 | 3.25E-10 | 977323 | 4.06E-05 | 40 |
| **MI** |  |  |  |  |  |  |  |  |  |
| **SNP** | **EA** | **Position** | **EAF** | **BETA** | **SE** | **P** | **N** | **R2** | **F** |
| rs10176176 | T | 85762048 | 0.48 | 0.064 | 0.007 | 8.73E-18 | 58825 | 2.04E-03 | 120 |
| rs10404176 | G | 17832302 | 0.46 | 0.057 | 0.008 | 5.79E-12 | 58825 | 1.61E-03 | 95 |
| rs10455872 | G | 161010118 | 0.06 | 0.312 | 0.016 | 1.08E-82 | 58825 | 1.18E-02 | 700 |
| rs10774625 | G | 111910219 | 0.53 | -0.079 | 0.008 | 2.68E-23 | 58825 | 3.13E-03 | 185 |
| rs10841443a | G | 20220033 | 0.65 | 0.055 | 0.008 | 8.77E-12 | 58825 | 1.38E-03 | 81 |
| rs10857147a | T | 81181072 | 0.27 | 0.054 | 0.009 | 1.97E-10 | 58825 | 1.16E-03 | 68 |
| rs10947786 | A | 39156410 | 0.21 | -0.069 | 0.009 | 5.87E-14 | 58825 | 1.57E-03 | 93 |
| rs11057837 | T | 125311720 | 0.10 | 0.077 | 0.013 | 3.57E-09 | 58825 | 1.07E-03 | 63 |
| rs11099493 | G | 82587050 | 0.30 | -0.054 | 0.008 | 4.87E-11 | 58825 | 1.22E-03 | 72 |
| rs112374545 | T | 11189272 | 0.11 | -0.105 | 0.012 | 2.18E-17 | 58825 | 2.16E-03 | 127 |
| rs113716316 | A | 27928640 | 0.07 | -0.088 | 0.016 | 4.36E-08 | 58825 | 9.55E-04 | 56 |
| rs11556924 | T | 129663496 | 0.34 | -0.063 | 0.008 | 5.51E-14 | 58825 | 1.76E-03 | 104 |
| rs11591147 | T | 55505647 | 0.02 | -0.294 | 0.036 | 1.80E-16 | 58825 | 2.71E-03 | 160 |
| rs11617955a | A | 110818102 | 0.11 | -0.094 | 0.012 | 4.46E-14 | 58825 | 1.69E-03 | 100 |
| rs11619113a | G | 110918660 | 0.12 | 0.067 | 0.012 | 7.35E-09 | 58825 | 9.31E-04 | 55 |
| rs11652894 | T | 17952439 | 0.56 | -0.046 | 0.008 | 4.87E-09 | 58825 | 1.05E-03 | 62 |
| rs1169288 | C | 121416650 | 0.33 | 0.056 | 0.008 | 1.75E-12 | 58825 | 1.40E-03 | 82 |
| rs117733303 | G | 160922870 | 0.02 | 0.409 | 0.033 | 2.19E-34 | 58825 | 7.22E-03 | 428 |
| rs12212146 | C | 161125454 | 0.06 | -0.101 | 0.017 | 4.90E-09 | 58825 | 1.16E-03 | 68 |
| rs12693302 | A | 183211443 | 0.61 | -0.046 | 0.008 | 2.53E-09 | 58825 | 9.97E-04 | 59 |
| rs12740374 | T | 109817590 | 0.21 | -0.106 | 0.009 | 8.39E-32 | 58825 | 3.74E-03 | 221 |
| rs12743267 | T | 95249306 | 0.23 | -0.050 | 0.009 | 1.06E-08 | 58825 | 9.04E-04 | 53 |
| rs12897285 | C | 100137162 | 0.17 | -0.061 | 0.010 | 2.93E-09 | 58825 | 1.04E-03 | 61 |
| rs12906125 | A | 91427612 | 0.31 | 0.075 | 0.008 | 6.52E-19 | 58825 | 2.41E-03 | 142 |
| rs13324341 | T | 138070901 | 0.16 | 0.064 | 0.010 | 4.17E-10 | 58825 | 1.08E-03 | 63 |
| rs13402621 | C | 43458611 | 0.22 | 0.055 | 0.009 | 1.14E-09 | 58825 | 1.03E-03 | 61 |
| rs1412445 | T | 91002804 | 0.35 | 0.073 | 0.008 | 1.41E-20 | 58825 | 2.41E-03 | 142 |
| rs16854041 | G | 216287497 | 0.26 | -0.049 | 0.009 | 1.45E-08 | 58825 | 8.95E-04 | 53 |
| rs16986953 | A | 19942473 | 0.08 | 0.087 | 0.013 | 4.15E-11 | 58825 | 1.15E-03 | 68 |
| rs17114046 | G | 56966350 | 0.09 | -0.102 | 0.013 | 3.41E-15 | 58825 | 1.70E-03 | 100 |
| rs17115100 | T | 104591393 | 0.11 | -0.071 | 0.012 | 1.05E-09 | 58825 | 9.66E-04 | 57 |
| rs180803 | T | 24664732 | 0.02 | -0.145 | 0.026 | 1.56E-08 | 58825 | 9.12E-04 | 54 |
| rs1967604 | G | 110530324 | 0.70 | -0.054 | 0.008 | 1.20E-10 | 58825 | 1.23E-03 | 72 |
| rs2019090a | T | 103668962 | 0.67 | -0.071 | 0.008 | 2.39E-18 | 58825 | 2.20E-03 | 130 |
| rs2107595 | A | 19049388 | 0.18 | 0.054 | 0.010 | 1.37E-08 | 58825 | 8.61E-04 | 51 |
| rs2327426 | C | 134202690 | 0.30 | -0.074 | 0.008 | 2.64E-20 | 58825 | 2.30E-03 | 136 |
| rs2452009 | G | 95495908 | 0.30 | -0.048 | 0.008 | 5.80E-09 | 58825 | 9.51E-04 | 56 |
| rs2505083 | C | 30335122 | 0.41 | 0.047 | 0.008 | 7.22E-10 | 58825 | 1.06E-03 | 62 |
| rs2519093 | T | 136141870 | 0.18 | 0.085 | 0.010 | 4.56E-19 | 58825 | 2.19E-03 | 129 |
| rs259979 | T | 57750556 | 0.15 | 0.062 | 0.011 | 1.24E-08 | 58825 | 9.45E-04 | 56 |
| rs2681472 | G | 90008959 | 0.18 | 0.067 | 0.009 | 1.26E-12 | 58825 | 1.33E-03 | 79 |
| rs2760740 | C | 2020989 | 0.30 | 0.052 | 0.008 | 1.19E-10 | 58825 | 1.12E-03 | 66 |
| rs2820309 | G | 201834944 | 0.32 | 0.043 | 0.008 | 4.19E-08 | 58825 | 8.21E-04 | 48 |
| rs28429551a | A | 139243334 | 0.76 | 0.057 | 0.010 | 1.72E-08 | 58825 | 1.19E-03 | 70 |
| rs28451064 | A | 35593827 | 0.12 | 0.117 | 0.012 | 2.96E-22 | 58825 | 2.98E-03 | 176 |
| rs2891168 | G | 22098619 | 0.48 | 0.188 | 0.007 | 1.80E-131 | 58825 | 1.77E-02 | 1062 |
| rs2954021 | G | 126482077 | 0.50 | -0.054 | 0.007 | 2.75E-13 | 58825 | 1.45E-03 | 86 |
| rs2972149a | A | 227092150 | 0.48 | 0.042 | 0.007 | 1.64E-08 | 58825 | 8.74E-04 | 51 |
| rs3127580 | T | 160710851 | 0.14 | 0.080 | 0.011 | 1.11E-13 | 58825 | 1.51E-03 | 89 |
| rs3128759 | T | 31885930 | 0.64 | 0.046 | 0.008 | 1.97E-08 | 58825 | 9.64E-04 | 57 |
| rs34633566a | C | 46306719 | 0.35 | 0.043 | 0.008 | 2.86E-08 | 58825 | 8.53E-04 | 50 |
| rs35700460 | G | 222811407 | 0.67 | 0.087 | 0.008 | 2.47E-24 | 58825 | 3.29E-03 | 194 |
| rs3772800 | C | 124438586 | 0.16 | 0.062 | 0.010 | 8.24E-10 | 58825 | 1.04E-03 | 61 |
| rs3918226 | T | 150690176 | 0.07 | 0.114 | 0.016 | 4.31E-13 | 58825 | 1.68E-03 | 99 |
| rs429358 | C | 45411941 | 0.14 | 0.108 | 0.011 | 4.21E-22 | 58825 | 2.80E-03 | 165 |
| rs433903 | G | 154000612 | 0.84 | 0.063 | 0.011 | 1.74E-08 | 58825 | 1.06E-03 | 63 |
| rs56210800a | G | 124472592 | 0.12 | 0.062 | 0.011 | 1.95E-08 | 58825 | 8.30E-04 | 49 |
| rs589655a | G | 44745315 | 0.11 | -0.098 | 0.011 | 1.52E-17 | 58825 | 1.86E-03 | 109 |
| rs6102343 | A | 39924279 | 0.24 | 0.049 | 0.009 | 1.58E-08 | 58825 | 8.78E-04 | 52 |
| rs62076439 | T | 47404628 | 0.34 | 0.048 | 0.008 | 1.47E-09 | 58825 | 1.04E-03 | 61 |
| rs6694258 | A | 154428505 | 0.56 | -0.047 | 0.007 | 4.09E-10 | 58825 | 1.07E-03 | 63 |
| rs6761276 | C | 113832312 | 0.57 | -0.042 | 0.008 | 2.84E-08 | 58825 | 8.76E-04 | 52 |
| rs6841581 | A | 148401190 | 0.15 | 0.065 | 0.010 | 2.74E-10 | 58825 | 1.08E-03 | 64 |
| rs7025486 | A | 124422403 | 0.26 | 0.047 | 0.008 | 4.41E-08 | 58825 | 8.33E-04 | 49 |
| rs7137258 | A | 54512164 | 0.07 | 0.114 | 0.016 | 1.72E-12 | 58825 | 1.61E-03 | 95 |
| rs7173743 | C | 79141784 | 0.45 | -0.064 | 0.007 | 1.97E-17 | 58825 | 2.00E-03 | 118 |
| rs72689147 | T | 156639888 | 0.18 | -0.068 | 0.010 | 7.39E-13 | 58825 | 1.38E-03 | 81 |
| rs72743461 | A | 67441750 | 0.21 | -0.061 | 0.009 | 4.65E-11 | 58825 | 1.24E-03 | 73 |
| rs72926767a | G | 203796814 | 0.12 | 0.112 | 0.012 | 1.06E-21 | 58825 | 2.57E-03 | 152 |
| rs73078367 | T | 48708347 | 0.11 | -0.080 | 0.012 | 4.65E-11 | 58825 | 1.25E-03 | 74 |
| rs73225849 | C | 22047223 | 0.06 | 0.097 | 0.017 | 5.47E-09 | 58825 | 1.02E-03 | 60 |
| rs748431 | T | 14928077 | 0.60 | -0.042 | 0.008 | 3.84E-08 | 58825 | 8.39E-04 | 49 |
| rs765549 | G | 19866631 | 0.29 | -0.047 | 0.008 | 1.85E-08 | 58825 | 9.05E-04 | 53 |
| rs9349379 | G | 12903957 | 0.41 | 0.125 | 0.008 | 2.07E-60 | 58825 | 7.60E-03 | 450 |
| rs9486719 | A | 97060124 | 0.20 | -0.057 | 0.009 | 6.80E-10 | 58825 | 1.03E-03 | 61 |
| rs9591012 | A | 33058333 | 0.34 | -0.044 | 0.008 | 3.68E-08 | 58825 | 8.61E-04 | 51 |
| rs9640375 | T | 139737344 | 0.22 | -0.058 | 0.009 | 2.39E-10 | 58825 | 1.16E-03 | 68 |
| rs9865841 | G | 135907213 | 0.75 | 0.057 | 0.009 | 2.06E-10 | 58825 | 1.22E-03 | 72 |
| rs9945890 | T | 46515916 | 0.63 | 0.045 | 0.008 | 1.65E-08 | 58825 | 9.33E-04 | 55 |
| **Stroke** |  |  |  |  |  |  |  |  |  |
| **SNP** | **EA** | **Position** | **EAF** | **BETA** | **SE** | **P** | **N** | **R2** | **F** |
| rs1052053 | G | 156202173 | 0.40 | -0.062 | 0.008 | 2.70E-14 | 446696 | 1.87E-03 | 835 |
| rs10776752 | T | 113044328 | 0.16 | 0.072 | 0.013 | 2.50E-08 | 446696 | 1.36E-03 | 609 |
| rs11191833 | A | 105619678 | 0.39 | -0.053 | 0.008 | 1.66E-10 | 446696 | 1.33E-03 | 593 |
| rs11957829 | G | 121515195 | 0.18 | -0.065 | 0.012 | 1.62E-08 | 446696 | 1.24E-03 | 552 |
| rs12445022 | A | 87575332 | 0.31 | 0.057 | 0.009 | 1.05E-10 | 446696 | 1.41E-03 | 629 |
| rs16896398a | T | 43262704 | 0.34 | 0.048 | 0.008 | 1.30E-08 | 446696 | 1.02E-03 | 455 |
| rs2107595 | A | 19049388 | 0.22 | 0.073 | 0.010 | 4.86E-14 | 446696 | 1.82E-03 | 813 |
| rs2129977 | G | 111712432 | 0.68 | -0.073 | 0.009 | 9.40E-16 | 446696 | 2.31E-03 | 1032 |
| rs3184504 | C | 111884608 | 0.55 | -0.062 | 0.009 | 8.63E-12 | 446696 | 1.91E-03 | 853 |
| rs35436 | T | 115554523 | 0.38 | -0.046 | 0.008 | 2.86E-08 | 446696 | 1.01E-03 | 451 |
| rs42039 | T | 92244422 | 0.23 | -0.060 | 0.011 | 7.32E-09 | 446696 | 1.29E-03 | 578 |
| rs4959130 | A | 1356916 | 0.14 | 0.078 | 0.013 | 1.42E-09 | 446696 | 1.44E-03 | 646 |
| rs6825454 | C | 155501188 | 0.30 | 0.052 | 0.009 | 2.61E-09 | 446696 | 1.13E-03 | 507 |
| rs7859727 | T | 22102165 | 0.53 | 0.049 | 0.008 | 4.22E-10 | 446696 | 1.22E-03 | 544 |
| rs8103309 | C | 11174935 | 0.35 | -0.050 | 0.009 | 3.40E-08 | 446696 | 1.13E-03 | 507 |
| rs880315 | C | 10796866 | 0.40 | 0.053 | 0.008 | 3.62E-10 | 446696 | 1.33E-03 | 596 |
| rs9316222 | C | 47214690 | 0.78 | 0.060 | 0.010 | 4.31E-10 | 446696 | 1.23E-03 | 552 |
| **IS** |  |  |  |  |  |  |  |  |  |
| **SNP** | **EA** | **Position** | **EAF** | **BETA** | **SE** | **P** | **N** | **R2** | **F** |
| rs1052053 | G | 156202173 | 0.40 | -0.058 | 0.009 | 4.48E-11 | 440328 | 1.59E-03 | 703 |
| rs1053007 | G | 10754400 | 0.65 | 0.048 | 0.009 | 3.58E-08 | 440328 | 1.04E-03 | 460 |
| rs11957829 | G | 121515195 | 0.18 | -0.072 | 0.012 | 7.51E-09 | 440328 | 1.50E-03 | 662 |
| rs12445022 | A | 87575332 | 0.31 | 0.061 | 0.010 | 1.28E-10 | 440328 | 1.57E-03 | 694 |
| rs17035646 | A | 10796547 | 0.41 | 0.054 | 0.009 | 1.34E-09 | 440328 | 1.38E-03 | 611 |
| rs2005108 | T | 102770353 | 0.13 | 0.080 | 0.015 | 3.33E-08 | 440328 | 1.43E-03 | 630 |
| rs2107595 | A | 19049388 | 0.23 | 0.076 | 0.010 | 9.25E-14 | 440328 | 2.02E-03 | 890 |
| rs3184504 | C | 111884608 | 0.55 | -0.075 | 0.010 | 2.17E-14 | 440328 | 2.79E-03 | 1234 |
| rs35436 | T | 115554523 | 0.38 | -0.050 | 0.009 | 3.21E-08 | 440328 | 1.16E-03 | 510 |
| rs42039 | T | 92244422 | 0.23 | -0.066 | 0.011 | 6.55E-09 | 440328 | 1.51E-03 | 667 |
| rs4932370 | A | 91404705 | 0.33 | 0.052 | 0.009 | 2.88E-08 | 440328 | 1.20E-03 | 528 |
| rs4959130 | A | 1356916 | 0.14 | 0.083 | 0.014 | 2.83E-09 | 440328 | 1.64E-03 | 723 |
| rs6825454 | C | 155501188 | 0.31 | 0.056 | 0.009 | 7.43E-10 | 440328 | 1.36E-03 | 598 |
| rs6847935a | T | 111696651 | 0.33 | 0.078 | 0.010 | 3.50E-16 | 440328 | 2.70E-03 | 1192 |
| rs7304841 | C | 20577593 | 0.41 | -0.048 | 0.009 | 4.93E-08 | 440328 | 1.13E-03 | 498 |
| rs7859727 | T | 22102165 | 0.54 | 0.051 | 0.008 | 1.05E-09 | 440328 | 1.31E-03 | 579 |
| rs9526212 | G | 47225745 | 0.76 | 0.062 | 0.010 | 9.19E-10 | 440328 | 1.37E-03 | 606 |
| rs9909858 | C | 1570587 | 0.19 | 0.089 | 0.016 | 3.63E-08 | 440328 | 2.43E-03 | 1075 |

SNP, single-nucleotide polymorphism; EAF, effect allele frequency; EA, effect allele; BETA, beta. exposure; SE, standard error; P, the Significance level of cardiovascular disease; R² was calculated as follows: 2*BETA^2*EAF*(1-EAF). If the data of EAF is missing, R² was calculated as follows: BETA^2/(BETA^2+SE^2*N). The F-statistic for each SNP was calculated as follows: F =((N-2)*(R^2^/ (1− R^2^)), a Palindromic SNPs with intermediate allele frequencies (>0.3) were removed, AF, Atrial fibrillation; CAD, Coronary artery disease; HF, Heart failure; MI, Myocardial infarction; IS, Ischemic stroke. The rs11717013 was used proxy the rs4642101 in HF. The rs10423961，rs113722226，rs1958320，rs77036345，rs355788，rs6779146 were used proxy the rs10404176，rs112374545，rs12897285，rs180803，rs433903，rs9865841 in MI, respectively. The rs60460011 was used proxy the rs9909858 in MI.

**Supplementary Table 3** | Characteristics of SNPs associated with frozen shoulder.

| SNP | EA | Position | EAF | BETA | SE | P | N | R^2^ | F |
| --- | --- | --- | --- | --- | --- | --- | --- | --- | --- |
| rs12114409 | A | 64684147 | 0.04 | -0.366 | 0.076 | 1.38E-06 | 170583 | 1.83E-02 | 265 |
| rs13095891 | T | 48381826 | 0.04 | -0.333 | 0.070 | 1.92E-06 | 170583 | 1.71E-02 | 248 |
| rs1549105 | C | 31078470 | 0.69 | -0.133 | 0.029 | 4.74E-06 | 170583 | 1.53E-02 | 221 |
| rs1831635 | A | 85300270 | 0.64 | -0.132 | 0.028 | 2.56E-06 | 170583 | 1.60E-02 | 231 |
| rs1870830 | C | 158541597 | 0.19 | -0.167 | 0.035 | 1.49E-06 | 170583 | 1.71E-02 | 248 |
| rs2236308 | G | 23313182 | 0.43 | -0.127 | 0.028 | 3.99E-06 | 170583 | 1.58E-02 | 228 |
| rs2354767 | T | 72076433 | 0.07 | 0.276 | 0.053 | 2.22E-07 | 170583 | 2.06E-02 | 299 |
| rs2482077 | C | 117851672 | 0.44 | -0.126 | 0.027 | 3.69E-06 | 170583 | 1.56E-02 | 225 |
| rs28599891ab | G | 32452470 | 0.66 | 0.138 | 0.029 | 2.12E-06 | 170583 | 1.72E-02 | 248 |
| rs35464280c | T | 222424225 | 0.00 | 1.056 | 0.228 | 3.43E-06 | 170583 | 1.97E-02 | 286 |
| rs71382481 | T | 36024611 | 0.17 | 0.171 | 0.036 | 2.41E-06 | 170583 | 1.62E-02 | 234 |
| rs9330811 | G | 46362396 | 0.53 | 0.226 | 0.027 | 8.64E-17 | 170583 | 5.08E-02 | 761 |

SNP, single-nucleotide polymorphism; EAF, effect allele frequency; EA, effect allele; BETA, beta. exposure; SE, standard error; P, the Significance level of frozen shoulder; R² was calculated as follows: 2*BETA^2*EAF*(1-EAF). The F-statistic for each SNP was calculated as follows: F =((N-2)*(R^2^/ (1− R^2^)). a Palindromic SNPs with intermediate allele frequencies (MAF>0.3) were removed; b CAD, HF and MI don't have the rs28599891; c Stroke, IS and HF don't have the rs35464280; The rs7613052 was used proxy the rs1870830 in HF.

**Supplementary Table 4** | Heterogeneity and pleiotropy analysis in reverse MR analysis.

| **Outcome** | **MR Method** | **Cochran Q statistic** | **Egger intercept** | **Heterogeneity p_value** | **Pleiotropy p_value** |
| --- | --- | --- | --- | --- | --- |
| **AF** | MR Egger | 11.13 | 0.003 | 0.267 | 0.686 |
|  | IVW | 11.34 |  | 0.331 |  |
| **CAD** | MR Egger | 15.17 | -0.010 | 0.086 | 0.247 |
|  | IVW | 17.76 |  | 0.059 |  |
| **HF** | MR Egger | 6.96 | -0.007 | 0.541 | 0.519 |
|  | IVW | 7.42 |  | 0.594 |  |
| **MI** | MR Egger | 25.14 | -0.005 | 0.083 | 0.687 |
|  | IVW | 25.63 |  | 0.064 |  |
| **Stroke** | MR Egger | 29.60 | -0.014 | 0.056 | 0.472 |
|  | IVW | 32.03 |  | 0.079 |  |
| **IS** | MR Egger | 24.80 | -0.008 | 0.218 | 0.687 |
|  | IVW | 25.34 |  | 0.325 |  |

MR,mendelian randomization; IVW, Inverse variance weighted, AF, Atrial fibrillation; CAD, Coronary artery disease; HF, Heart failure; MI, Myocardial infarction; IS, Ischemic stroke.

**Supplementary Figure 1 |** SNP screening flow chart.

**
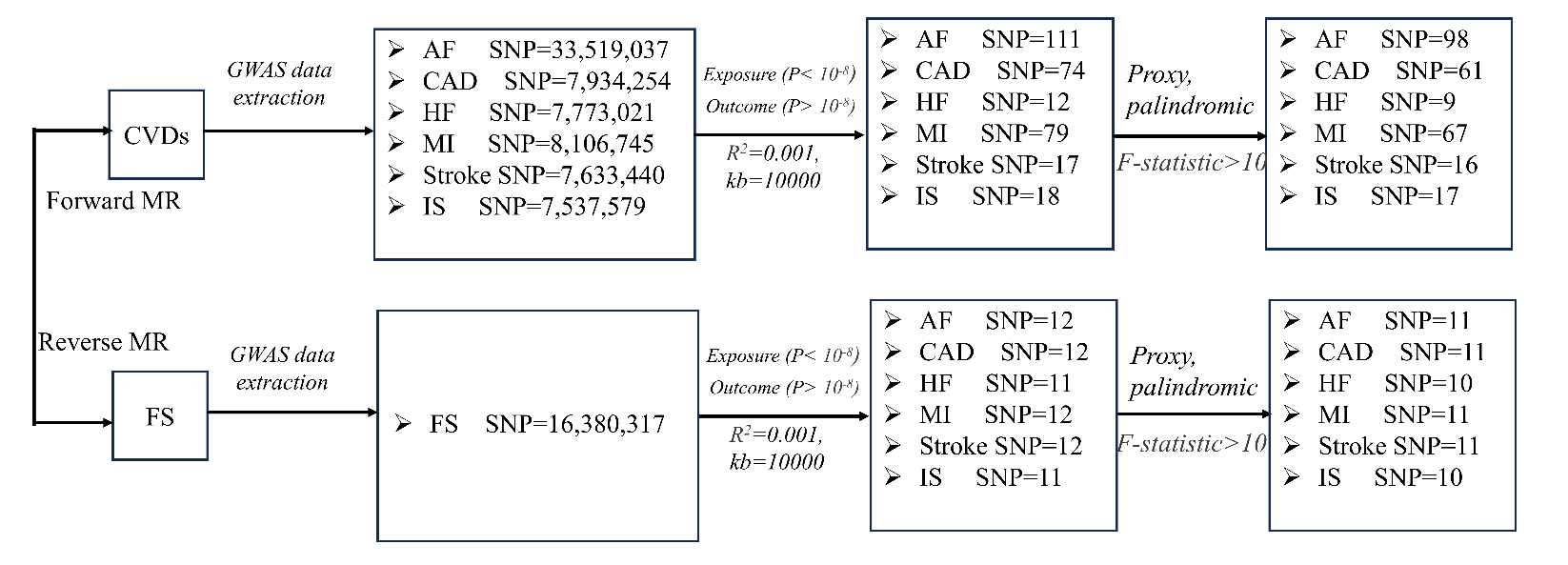
**

**Supplementary Figure 2 |** The forest plots for causal effect of cardiovascular disease on frozen shoulder.


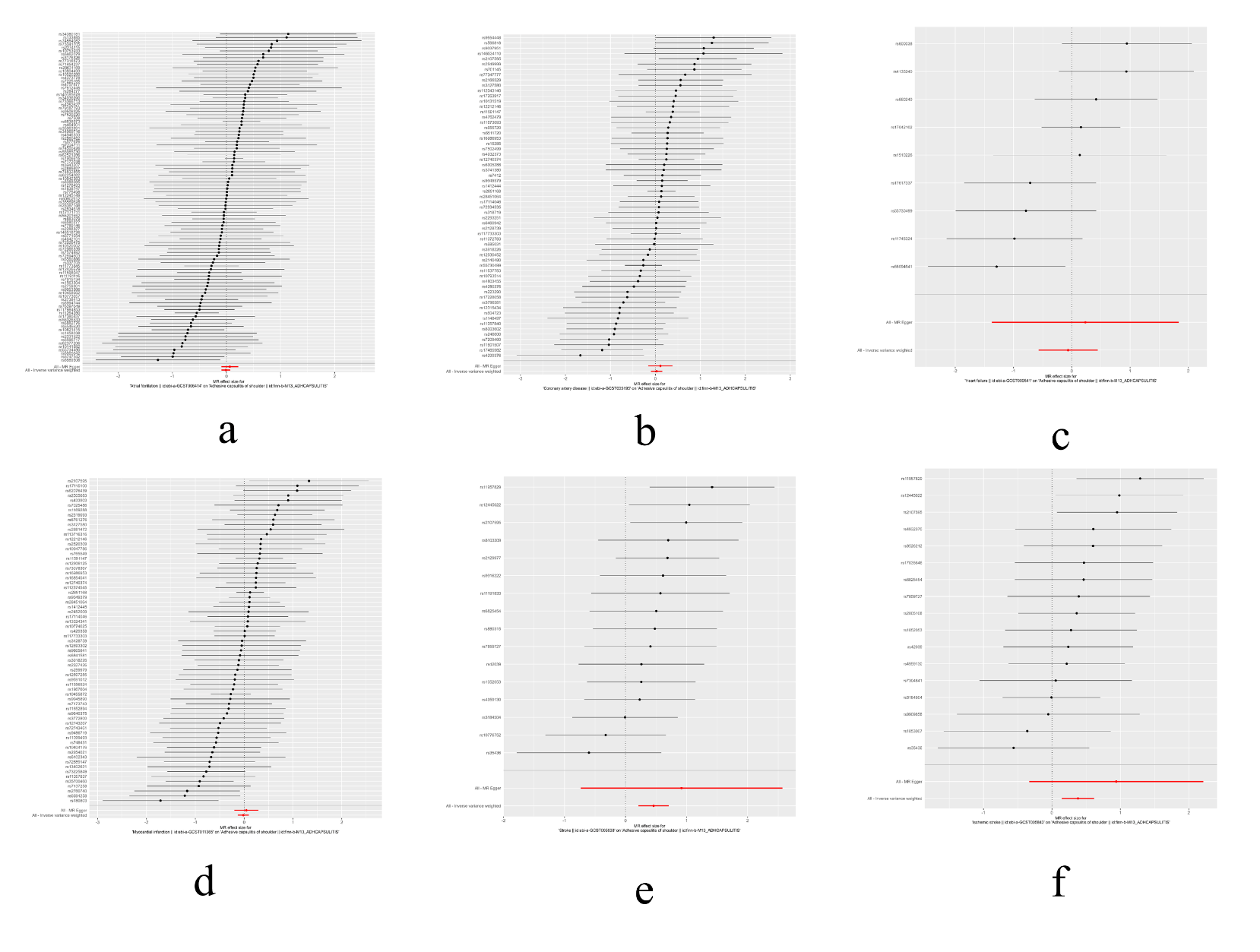


(a) Atrial fibrillation on Frozen shoulder, (b) Coronary artery disease on Frozen shoulder, (c) Heart failure on Frozen shoulder, (d) Myocardial infarction on Frozen shoulder, (e) Stroke on Frozen shoulder, (f) Ischemic stroke on Frozen shoulder.

**Supplementary Figure 3 |** Leave-one-out sensitivity analysis for causal effect of cardiovascular disease on frozen shoulder.


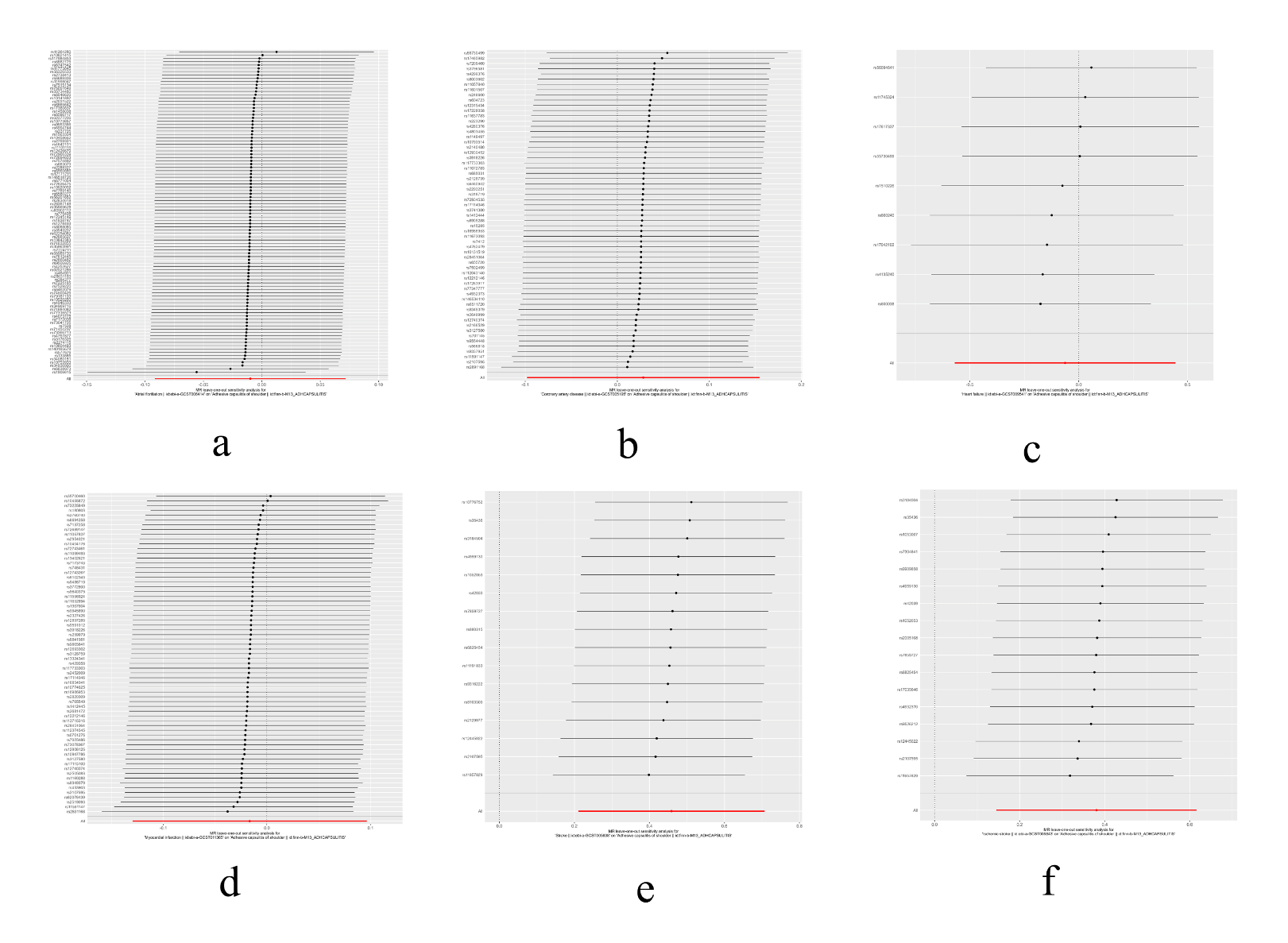


(a) Atrial fibrillation on Frozen shoulder, (b) Coronary artery disease on Frozen shoulder, (c) Heart failure on Frozen shoulder, (d) Myocardial infarction on Frozen shoulder, (e) Stroke on Frozen shoulder, (f) Ischemic stroke on Frozen shoulder.

**Supplementary Figure 4 |** The funnel chart for causal effect of cardiovascular disease on frozen shoulder.


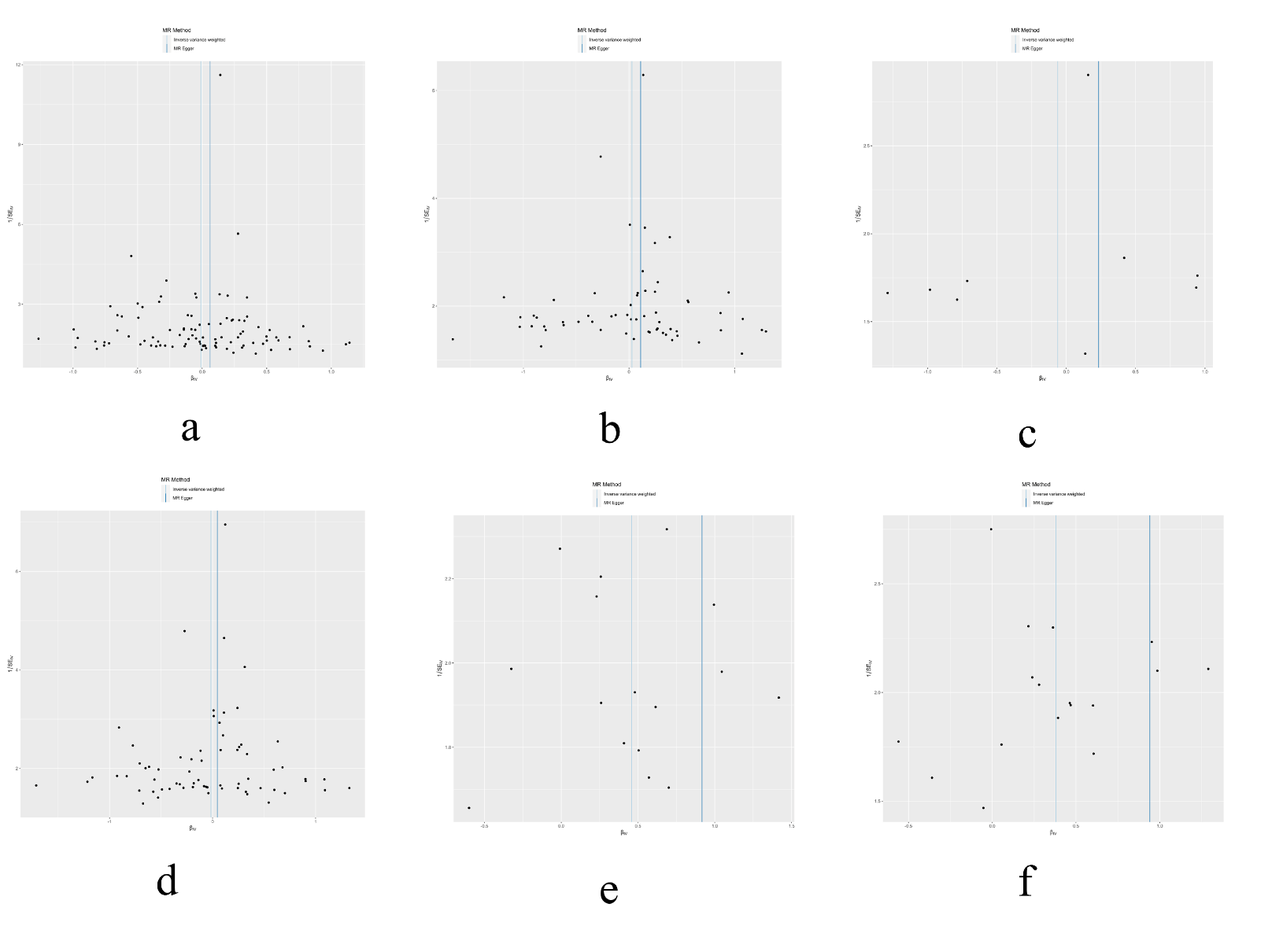


(a) Atrial fibrillation on Frozen shoulder, (b) Coronary artery disease on Frozen shoulder, (c) Heart failure on Frozen shoulder, (d) Myocardial infarction on Frozen shoulder, (e) stroke on Frozen shoulder, (f) Ischemic stroke on Frozen shoulder.

**Supplementary Figure 5 |** The scatter plots for causal effect of frozen shoulder on cardiovascular disease.

**
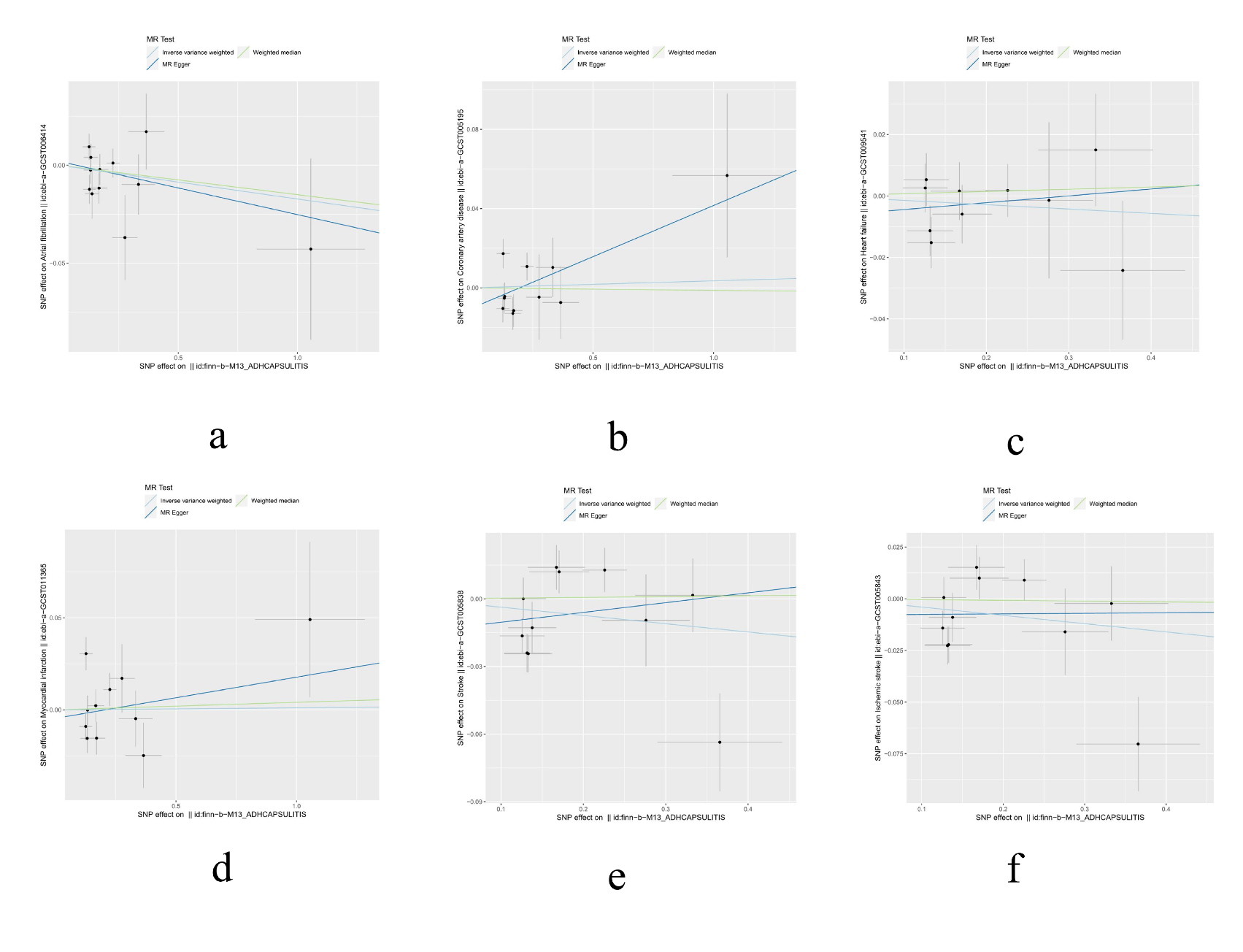
**

(a) Frozen shoulder on Atrial fibrillation, (b) Frozen shoulder on Coronary artery disease, (c) Frozen shoulder on Heart failure, (d) Frozen shoulder on Myocardial infarction, (e) Frozen shoulder on stroke, (f) Frozen shoulder on Ischemic stroke.

**Supplementary Figure 6 |** The forest plots for causal effect of frozen shoulder on cardiovascular disease.

**
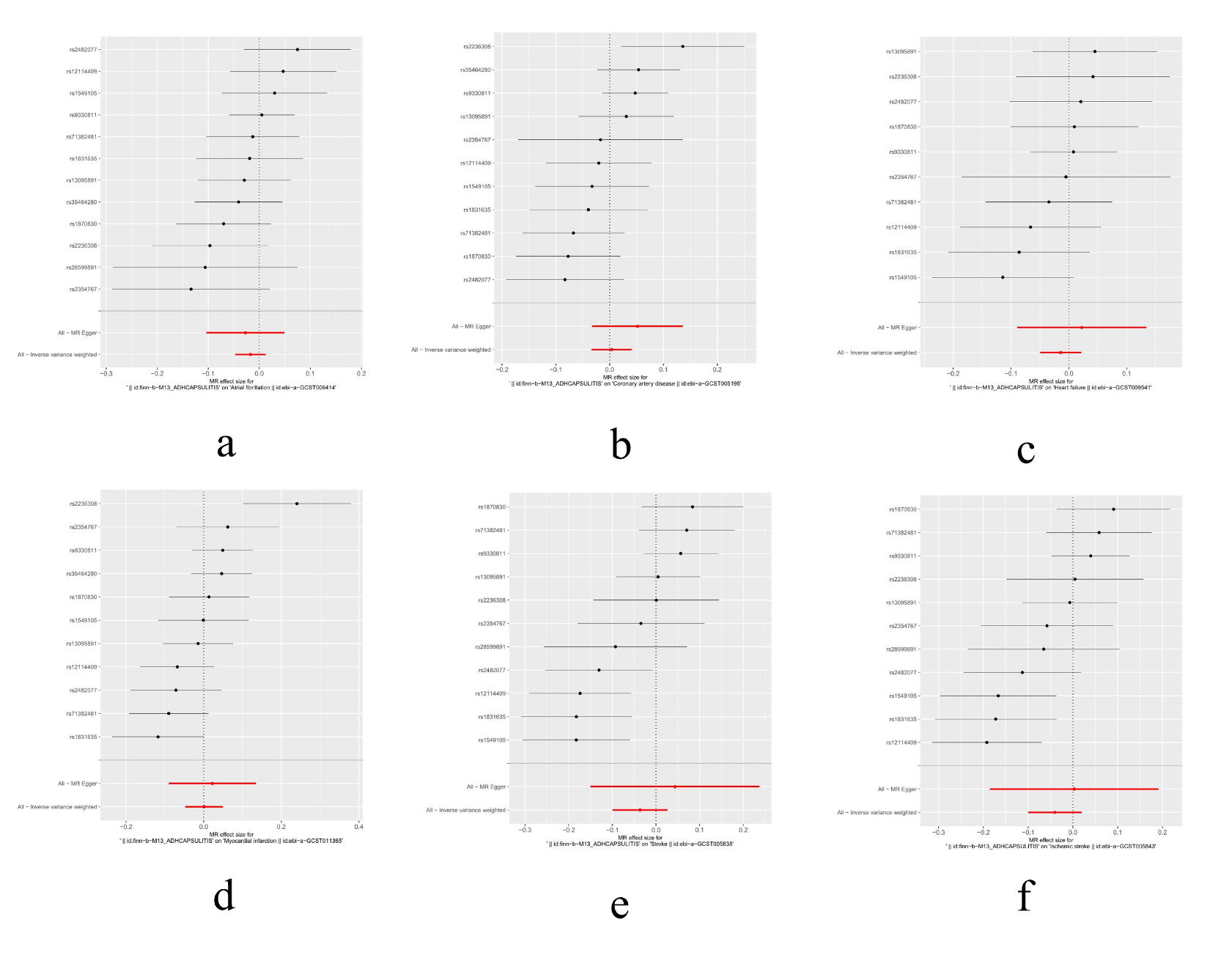
**

(a) Frozen shoulder on Atrial fibrillation, (b) Frozen shoulder on Coronary artery disease, (c) Frozen shoulder on Heart failure, (d) Frozen shoulder on Myocardial infarction, (e) Frozen shoulder on stroke, (f) Frozen shoulder on Ischemic stroke.

**Supplementary Figure 7 |** Leave-one-out sensitivity analysis for causal effect of frozen shoulder on cardiovascular disease.


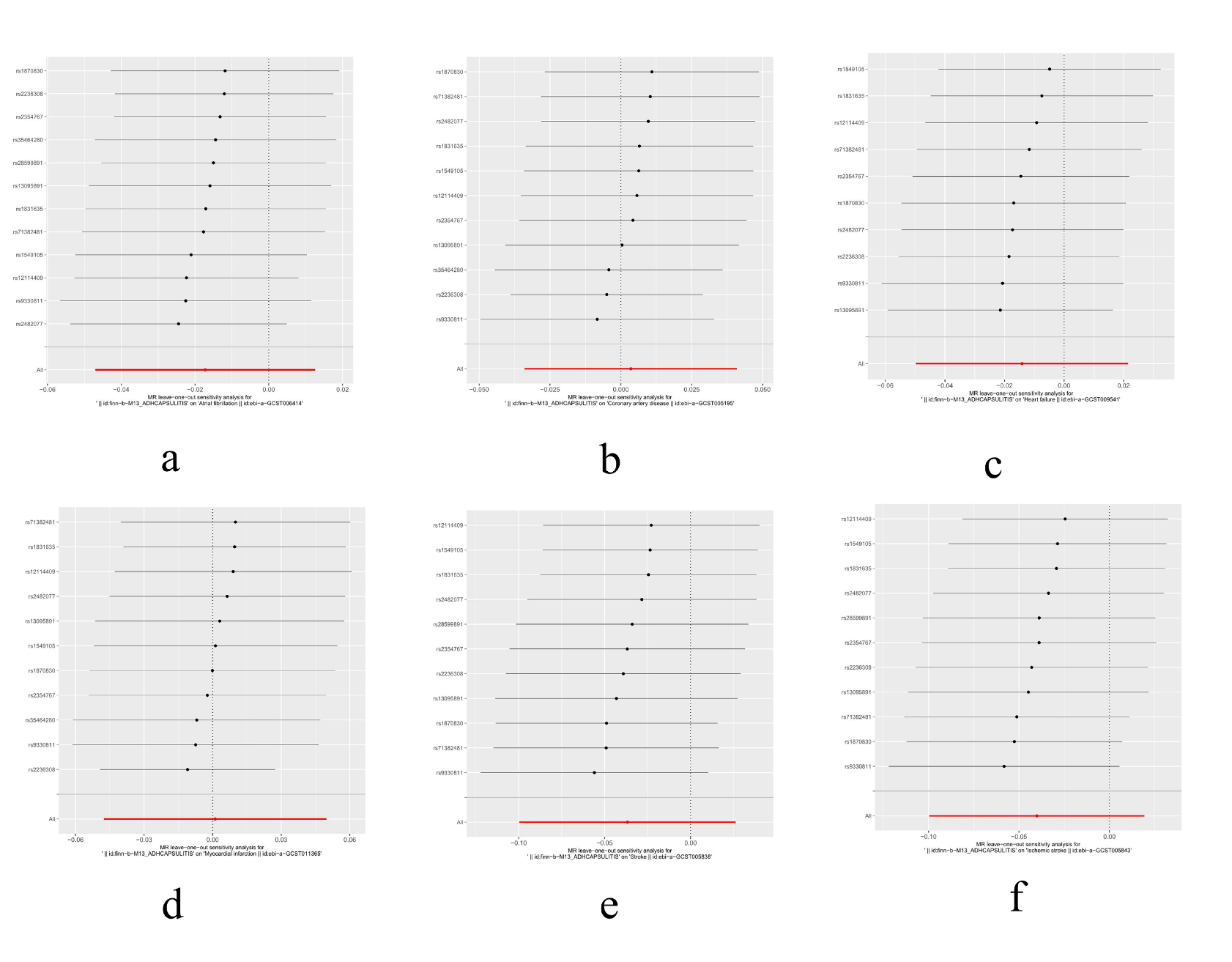


(a) Frozen shoulder on Atrial fibrillation, (b) Frozen shoulder on Coronary artery disease, (c) Frozen shoulder on Heart failure, (d) Frozen shoulder on Myocardial infarction, (e) Frozen shoulder on stroke, (f) Frozen shoulder on Ischemic stroke.

**Supplementary Figure 8 |** The funnel chart for causal effect of frozen shoulder on cardiovascular disease.


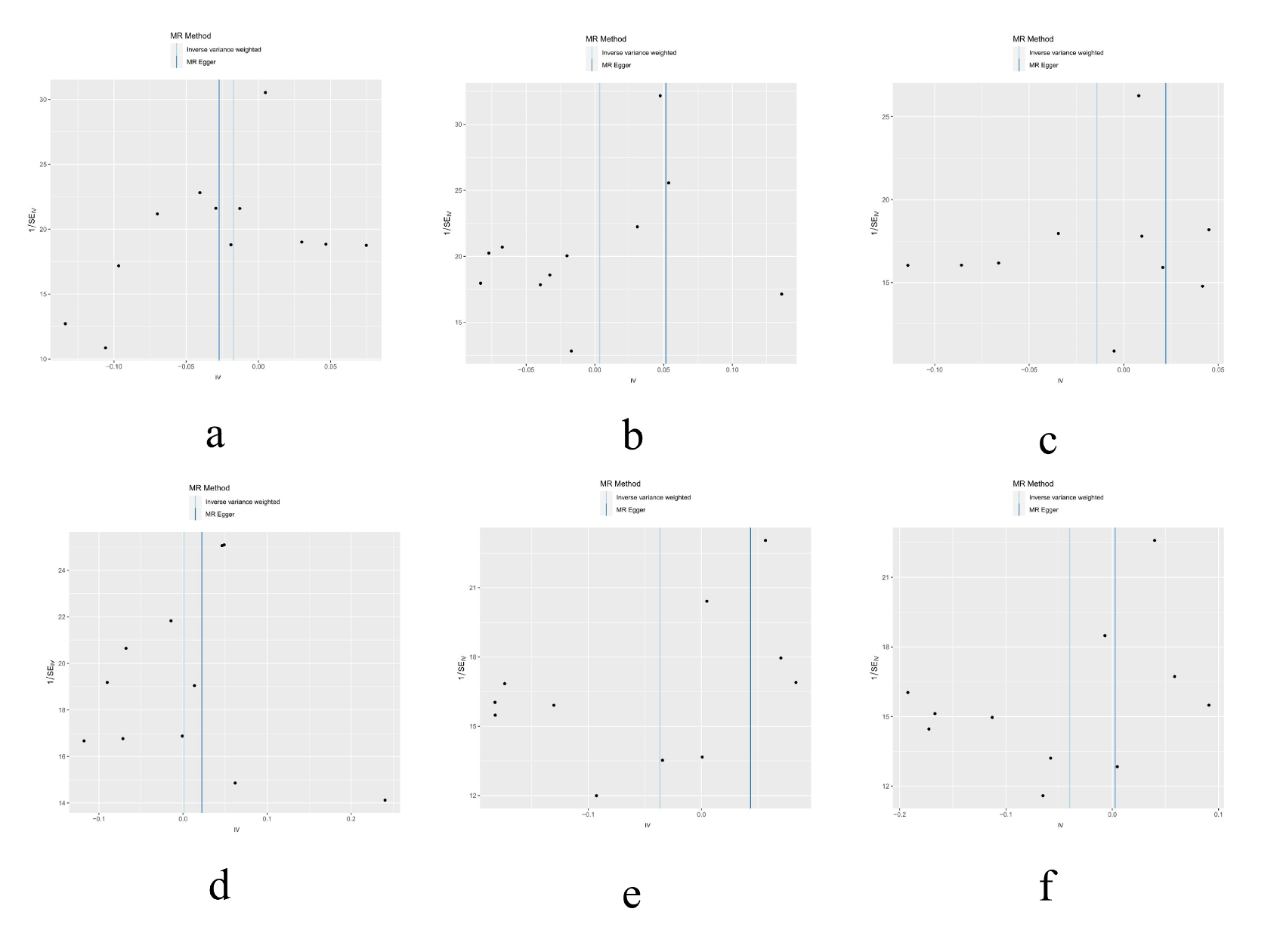


(a) Frozen shoulder on Atrial fibrillation, (b) Frozen shoulder on Coronary artery disease, (c) Frozen shoulder on Heart failure, (d) Frozen shoulder on Myocardial infarction, (e) Frozen shoulder on stroke, (f) Frozen shoulder on Ischemic stroke.
